# Expression profiling analysis of long noncoding RNAs in a mouse model of ventilator‐induced lung injury indicating potential roles in inflammation

**DOI:** 10.1002/jcb.28446

**Published:** 2019-02-19

**Authors:** Nan‐Nan Zhang, Yi Zhang, Lu Wang, Jin‐Gen Xia, Shun‐Tao Liang, Yan Wang, Zhi‐Zhi Wang, Xu Huang, Min Li, Hui Zeng, Qing‐Yuan Zhan

**Affiliations:** ^1^ Center for Respiratory Diseases, China‐Japan Friendship Hospital Beijing China; ^2^ Department of Pulmonary and Critical Care Medicine China‐Japan Friendship Hospital Beijing China; ^3^ National Clinical Research Center for Respiratory Diseases Beijing China; ^4^ Graduate School of Peking Union Medical College, Chinese Academy of Medical Sciences Beijing China; ^5^ Beijing Key Laboratory of Emerging Infectious Diseases, Institute of Infectious Diseases, Beijing Ditan Hospital, Capital Medical University Beijing China

**Keywords:** inflammation, long noncoding RNA, RNA sequencing, ventilator‐induced lung injury

## Abstract

The key regulators of inflammation underlying ventilator‐induced lung injury (VILI) remain poorly defined. Long noncoding RNAs (lncRNAs) have been implicated in the inflammatory response of many diseases; however, their roles in VILI remain unclear. We, therefore, performed transcriptome profiling of lncRNA and messenger RNA (mRNA) using RNA sequencing in lungs collected from mice model of VILI and control groups. Gene expression was analyzed through RNA sequencing and quantitative reverse transctiption polymerase chain reaction. A comprehensive bioinformatics analysis was used to characterize the expression profiles and relevant biological functions and for multiple comparisons among the controls and the injury models at different time points. Finally, lncRNA‐mRNA coexpression networks were constructed and dysregulated lncRNAs were analyzed functionally. The mRNA transcript profiling, coexpression network analysis, and functional analysis of altered lncRNAs indicated enrichment in the regulation of immune system/inflammation processes, response to stress, and inflammatory pathways. We identified the lncRNA Gm43181 might be related to lung damage and neutrophil activation via chemokine receptor chemokine (C‐X‐C) receptor 2. In summary, our study provides an identification of aberrant lncRNA alterations involved in inflammation upon VILI, and lncRNA‐mediated regulatory patterns may contribute to VILI inflammation.

## Graphical abstract

We performed transcriptome profiling of long noncoding RNA (lncRNA) and messenger RNA using RNA sequencing in lungs collected from mice model of the ventilator‐induced lung injury (VILI) and control groups. Our study provides an identification of aberrant lncRNA alterations involved in inflammation upon VILI, and lncRNA‐mediated regulatory patterns may contribute to VILI inflammation.

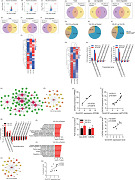

## 1 INTRODUCTION

Mechanical ventilation (MV) is widely used as a life‐saving option, especially in acute respiratory distress syndrome (ARDS) patients.^1^ However, despite optimizing ventilator parameters or the therapeutic modalities, there is increasing evidence that MV can cause or worsen acute and chronic damage to the lung, which is referred to as ventilator‐induced lung injury (VILI).^2^, ^3^

In addition to injury from the mechanical stretch, the subsequent overwhelming inflammation in the lungs is an important factor in promoting pulmonary damage.^3^, ^4^, ^5^, ^6^ Thus, reducing the inflammatory response could go a long way toward alleviating lung injury from MV.^7^, ^8^, ^9^ To date, although many efforts have been undertaken to explain the underlying mechanisms, the key regulators of inflammation that contribute to VILI remain poorly defined. Considering the role of inflammation in VILI development, improved understanding of the molecular mechanisms that promote the inflammatory response during VILI are extremely critical for novel therapeutic targets or effective prevention.

Following MV, the expression of many messenger RNAs (mRNAs) is markedly altered in the lungs and blood, and many candidate genes associated with inflammation have emerged.^10^, ^12^ Furthermore, there is now increasing evidence that numerous small noncoding RNAs, such as microRNA (miRNA), could play important roles in regulating inflammation.^13^ In fact, miRNA profiling studies in VILI have identified some miRNAs that are involved in regulating inflammation.^14^ Significantly, a large percentage of the transcriptome has been now identified as long noncoding RNA (lncRNA) by RNA sequencing (RNA‐Seq),^15^, ^16^ and these are emerging as crucial versatile regulators involved in various biological processes (BPs).^17^, ^18^ Increasingly, studies suggest that lncRNAs participate extensively in many inflammatory and immunologic diseases, like rheumatoid arthritis,^19^ asthma,^20^ or ARDS.^21^ However, the role of lncRNA in the inflammation underlying VILI pathogenesis has rarely been reported.

The potential role of lncRNAs in VILI was first reported by Xu et al,^22^ who identified 104 lncRNAs that were differentially expressed in the lungs of a VILI rat model. They also revealed that the altered lncRNAs were related to apoptosis, angiogenesis, and neutrophil chemotaxis. The lncRNA Map2k3os was shown to promote the expression of stretch‐induced cytokines. These results point toward important inflammatory mechanisms underlying VILI. Notably, some recent reports have indicated the involvement of lncRNAs in the inflammatory process.^23^, ^24^ Thus, the important role of inflammation in VILI and these previous observations prompted us to investigate the role of lncRNAs in inflammation during VILI progression.

In this study, we utilized a mouse model of lung injury induced by high tidal volume MV and performed RNA‐Seq–based lncRNA profiling analysis of lung tissues. We aimed to screen the dysregulated lncRNAs in the lung during early and relatively late time intervals of lung inflammation of VILI to provide new insight into the lncRNA‐mediated regulatory network of inflammation progression.

## 2 MATERIALS AND METHODS

### 2.1 Animal model

Male C57BL/6 mice at 6 to 8 weeks (18‐20 g; the Peking University Laboratory Animal Center, Beijing, China) maintained under a standard environment were randomly divided into five groups (*n* = 8 per group). The VILI mouse model was established using MV with a high tidal volume (20 ml/kg) for 4 hours as reported.^25^, ^26^ Briefly, we anesthetized mice with an intraperitoneal injection of ketamine (80 ml/kg) and xylazine (10 mg/kg). Mice were then fixed and intubated with a 20‐gauge intravenous catheter (BD Biosciences, San Jose, CA). An animal ventilator (Inspira; Harvard Apparatus, Holliston, MA) was connected using a volume‐controlled setting. Appropriate parameters were set as follows: tidal volume 20 ml/kg, respiratory frequency 40/min, positive end‐expiratory pressure, 0 cm H_2_O, FiO_2_ = 0.21. Anesthesia was maintained by administering the mixture of ketamine and xylazine; muscle relaxation was implemented and maintained with pancuronium (2 mg·kg^−1^·h^−1^). The control group comprised of intubated and nonventilated mice. A heating pad was used to maintain normothermia at 37°C throughout MV. Mice were allowed to recover and were exsanguinated under anesthesia at 0, 3, 6, 24 hours following 4 hours MV or control procedure. We cut the carotid artery and collected blood for the arterial blood gas analysis (Radiometer ABL; Radiometer, Carlsbad, CA) at the indicated time points. All animal procedures were approved by the Animal Ethics Committee of the China‐Japan Friendship Hospital (no. 13007). Experiments were performed in accordance with the *National Guidelines of Laboratory Animal Care* principles.

### 2.2 Bronchoalveolar lavage fluid collection and processing

Mice were killed by exsanguination at the indicated time points and bronchoalveolar lavage fluid (BALF) was collected.^27^ In short, the trachea was exposed, cannulated, and lavaged three times with 750 μL saline solution. BALF was filtered, centrifuged, and the supernatants were stored at –80°C until further analysis. For collecting BALF cells, a total of three repetitive perfusions were batched together and centrifuged. The BALF cells were then prepared for estimating the BALF protein concentration using a bicinchoninic acid protein assay, according to the manufacturer's instructions (Thermo Fisher Scientific, Waltham, MA).

### 2.3 Flow cytometry analysis

Mouse lungs were minced to single cell suspensions and filtered through 70‐μm cell strainers (BD Biosciences, San Jose, CA) as previously described.^9^, ^28^ The erythrocytes were lysed using red blood cell lysis buffer (BD Biosciences) and washed with phosphate‐buffered saline. Cells were labeled with monoclonal antibodies including anti‐mouse CD45‐FITC, Gr‐1‐PE, CD11b‐PerCP, CD48‐APC, and counting beads (BD Biosciences) was added. Matched isotype antibodies were used as negative controls. All samples were analyzed using a FACSCalibur cytometer (BD Biosciences) and CellQuest software, and data were analyzed using FlowJo software (Tree Star, Ashland, OR).

### 2.4 Cell sorting

For functional analysis, neutrophils (Gr‐1^+^CD48^−^) in lungs were purified by staining with anti‐mouse Gr‐1‐PE and CD48‐APC antibody (BD Biosciences) from VILI 6 hours, VILI 0 hours, and control groups using fluorescence‐activated cell sorter (FACS) Aria II flow cytometer (BD Biosciences). The purity of the neutrophils was more than 95%.

### 2.5 Histopathology

For histological evaluation, lungs were removed, and inflated in 4% paraformaldehyde. For favorable fixation, the lungs were embedded in paraffin and subjected to hematoxylin and eosin staining using a standard procedure. Two independent pathologists performed blinded histologic evaluation of the lung injury using 10 random high‐power fields.^29^, ^30^

### 2.6 RNA isolation and sequencing

Total RNA was extracted from the lung samples using TRIzol (Invitrogen, Carlsbad, CA) according to the manufacturer's instructions. RNA purity, concentration, and integrity were determined using a NanoPhotometer spectrophotometer (Implen Inc., Westlake Village, CA), Qubit RNA Assay Kit in a Qubit 2.0 Fluorometer (Life Technologies, Carlsbad, CA), and an RNA Nano 6000 Assay Kit of the Bioanalyzer 2100 system (Agilent Technologies, Savage, MD), respectively. Whole transcriptome libraries and deep sequencing were performed by Novogene Bioinformatics Technology Cooperation (Beijing, China). Briefly, a total of 3 μg RNA per sample was used as input material. Sequencing libraries were generated using rRNA‐depleted RNA by following manufacturer's instructions for the NEBNext Ultra Directional RNA Library Prep Kit for Illumina (New England Biolabs, Ipswich, MA). The Agilent Bioanalyzer 2100 system was used to evaluate the purity and quantity of the products. RNA‐Seq was performed on an Illumina HiSeq. 2500 platform and 125‐bp paired‐end reads were generated. The RNA‐seq data set was deposited in Gene Expression Omnibus (https://www.ncbi.nlm.nih.gov/geo/) under the accession number GSE120080.

### 2.7 Quality control, read mapping, and transcriptome assembly

The FASTQ format of raw data was processed and clean high‐quality reads were obtained by removing reads containing adapters, poly‐N, and those of low‐quality from the raw data. The paired‐end clean reads were aligned to the reference genome using TopHat (v2.0.9, Johns Hopkins University). The mapped reads of each sample were assembled by both Scripture (beta2, Broad Institute)^31^ and Cufflinks (v2.1.1, the University of Washington)^32^ using a reference‐based approach.

### 2.8 Quantification and analysis of differential gene expression

Cuffdiff (v2.1.1, the University of Washington) provided fragments per kilobase of exon per million fragments (FPKM) calculations of both lncRNAs and coding genes in each sample.^32^ The Gene FPKMs were computed by summing the FPKMs of transcripts in each gene group, that is, FPKMs were mapped and calculated based on the length of the fragment and the read counts mapped to this fragment. Transcripts with *P* < 0.05 were determined as being differentially expressed.

### 2.9 Gene Ontology and Kyoto Encyclopedia of Genes and Genomes enrichment analysis

Gene Ontology (GO) annotations of differentially expressed genes were performed by the GOseq R package and the corrected *P* < 0.05 were considered significantly enriched.^33^ KOBAS software (Peking University, China) was used to test the enrichment pathways of the differentially expressed genes according to Kyoto Encyclopedia of Genes and Genomes (KEGG).^34^

### 2.10 Protein‐protein interactions network construction

The differentially expressed genes were mapped to the STRING database (ELIXIR, Hinxton, Cambridgeshire, UK) to construct a protein‐protein interactions (PPI) network and were visualized by Cytoscape software (Institute for Systems Biology, Seattle, Washington).

### 2.11 Functional and coexpression network of lncRNAs

The target genes of lncRNAs were predicted based on functional annotations of the associated mRNAs in *cis* and *trans*. We searched for potential *cis* mRNAs within 100 kb upstream and downstream of each lncRNA. The coexpressed mRNAs of lncRNAs with the Pearson correlation coefficients over 0.95 or under −0.95, and beyond 100 kb in genomic distance from the lncRNA or located on different chromosomes were defined as the *trans*‐regulated genes. The coexpression network was constructed based on the screening of lncRNAs‐mRNAs gene pairs (Pearson correlation coefficient, >0.95 or <−0.95; *P* < 0.05) using Cytoscape software. The dysregulated lncRNAs were annotated by GO term and pathway analysis.

### 2.12 Quantitative real‐time polymerase chain reaction

Total RNA was extracted from lung samples or neutrophils using TRIzol reagent (Invitrogen), and a high‐capacity RNA‐to‐cDNA Kit (Invitrogen) was used to synthesize the complementary DNA using manufacturer's instructions. The quantitative real‐time polymerase chain reaction (qRT‐PCR) for the expression of selected lncRNAs and mRNAs was performed in triplicate with SYBR Green Master Mix (Invitrogen). lncRNA and mRNAs expression was quantified relative to glyceraldehyde 3‐phosphate dehydrogenase expression as fold change using the $2^{-\Delta \Delta C_{t}}$ method.

### 2.13 Statistical analysis

Statistical analysis was performed using GraphPad Prism 7 software (San Diego, CA). Kolmogorov‐Smirnov test was used to test the normality of the data. Data analysis were performed using one‐way analysis of variance (ANOVA) or Kruskal‐Wallis ANOVA on ranks with the Bonferroni test to estimate the parameters for multiple comparisons. The correlations were assessed using the Pearson correlation test. Data are reported as mean ± standard deviation (SD) for normally distributed data or as median (interquartile range). For all comparisons, statistical significance was defined as *P* < 0.05.

## 3 RESULTS

### 3.1 VILI model induced by high tidal volume MV

**Figure 1:**
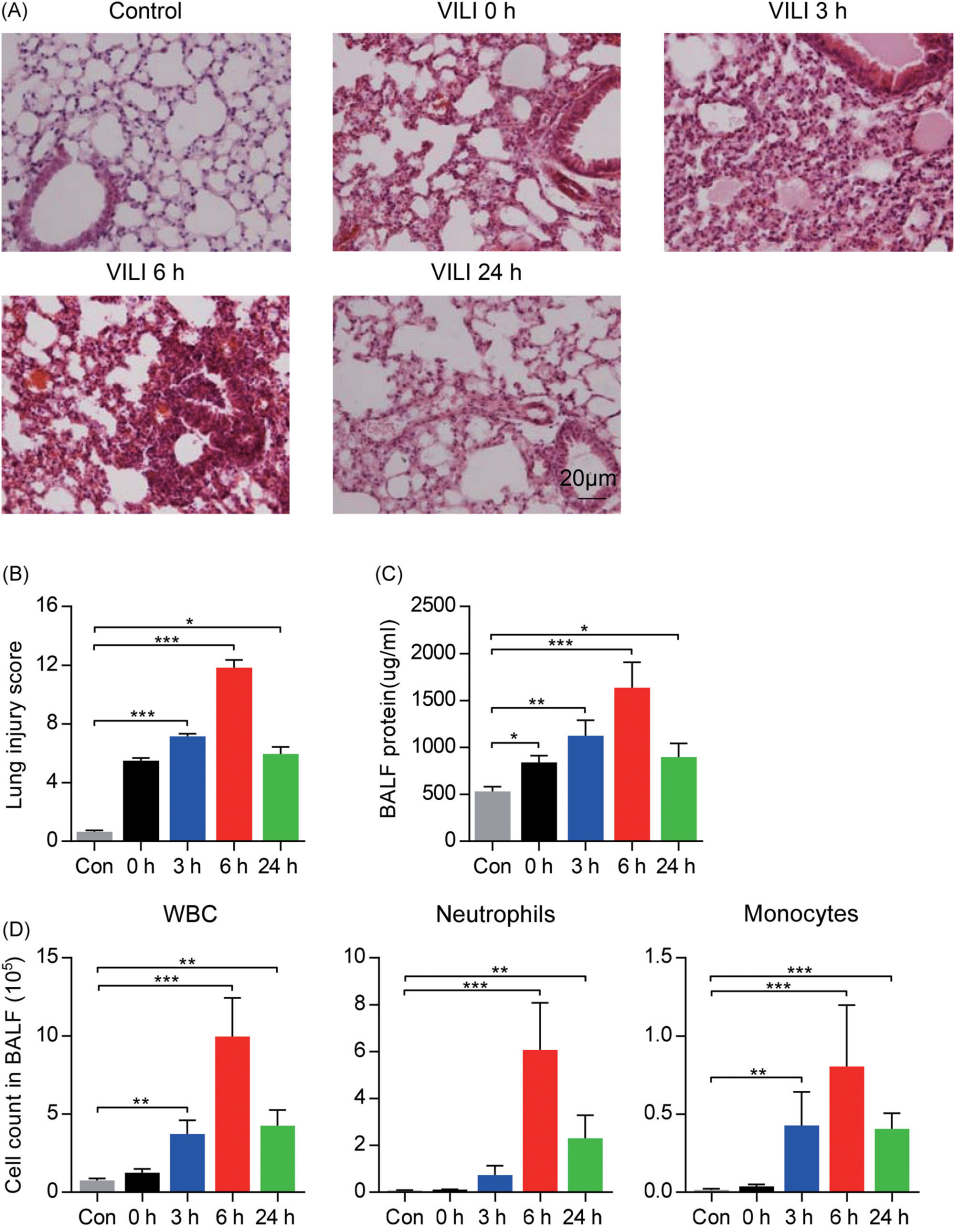
Identification of ventilator‐induced lung injury (VILI) model induced by high tidal volume mechanical ventilation. A, Lung tissues were stained with hematoxylin and eosin (HE) at indicated time points for histological evaluation. ×400, original magnification, *n* = 8 per group. B, The histopathologic lung injury scores are displayed. C, Total protein concentrations in BALF. D, Cell counts of white blood cells (WBC), neutrophils, and monocytes in BALF at indicated time points (*n* = 8 per group). Data are represented as the mean ± SD and analyzed by one‐way analysis of variance with the Bonferroni test. *P < 0.05, **P < 0.01, and ***P < 0.001. BALF, bronchoalveolar lavage fluid

Blood gas assessments demonstrated hypoventilation in the control group, which are mostly due to ventilation without the animal ventilator (Table 1). The mice exposed to high‐stretch ventilation displayed a significant disruption of the pulmonary architecture, including infiltration of inflammatory cells, alveolar hemorrhage and collapse, and increased alveolar wall thickness, when compared with the control mice (Figure 1A). The histological lesions were most severe in the 6 hours post‐MV group. The lung injury scores were markedly increased in VILI model animals compared with the control animals, with maximum injury seen at 6 hours post‐MV (Figure 1B). Arterial oxygen tension was maximally decreased at 6 hours post‐MV as well (Table 1).

**Table 1:** Arterial blood gas assessments

Parameters	Control	VILI 0 h	VILI 3 h	VILI 6 h	VILI 24 h
pH	7.43 ± 0.02	7.42 ± 0.29	7.4 ± 0.39	7.39 ± 0.31	7.41 ± 0.01
PaO_2_ (mm Hg)	79 ± 3.34	87 ± 6.37	83 ± 8.37	70 ± 2.51^a^	85 ± 5.21
PaCO_2_ (mm Hg)	55 ± 5.83	53 ± 3.34	54.4 ± 3.98	58 ± 4.63	52.1 ± 6.21
HCO_3_^−^ (mmol/L)	22.8 ± 0.60	22.5 ± 0.93	21.9 ± 1.13	21.5 ± 0.80	22.2 ± 1.04
Base excess (mmol/L)	−2.8 ± 1.33	−3.1 ± 1.21	−3.3 ± 0.83	−3.6 ± 0.91	−3.7 ± 0.97

Abbreviations: PaCO_2_, partial pressure of CO_2_; PaO_2_, partial pressure of O_2_; VILI, ventilator‐induced lung injury.

Data are expressed as mean ± SD.

^a^ Significantly different from control group using one‐way analysis of variance with the Bonferroni test (*P* < 0.05).

The quantification of BALF protein was also found to peak at VILI 6 hours post‐MV (Figure 1C). Further, a significant accumulation of inflammatory cells including white blood cells (WBC), neutrophils, and monocytes within BALF were detected by flow cytometry. A significant increment of these inflammatory cells in BALF was observed at 6 hours following the high‐stretch ventilator treatment (Figure 1D). Collectively, these results suggested that high tidal volume ventilation for 4 hours could successfully induce severe lung inflammation, which peaked at 6 hours after MV.

### 3.2 Expression of mRNAs and lncRNAs was altered by high‐stretch ventilation in the course of VILI

To identify novel molecular features of VILI, high‐throughput RNA‐Seq was carried out in a mouse model of VILI. We extracted and sequenced the total lung RNA from three VILI 0 hours mice (mice killed immediately after MV), three VILI 6 hours mice (time intervals 6 hours post‐MV), and three control mice, during early and relatively late time intervals of lung inflammation. Low‐quality reads and rRNA were depleted and the total clean reads were uniquely mapped to the genome (Table S1).

**Figure 2:**
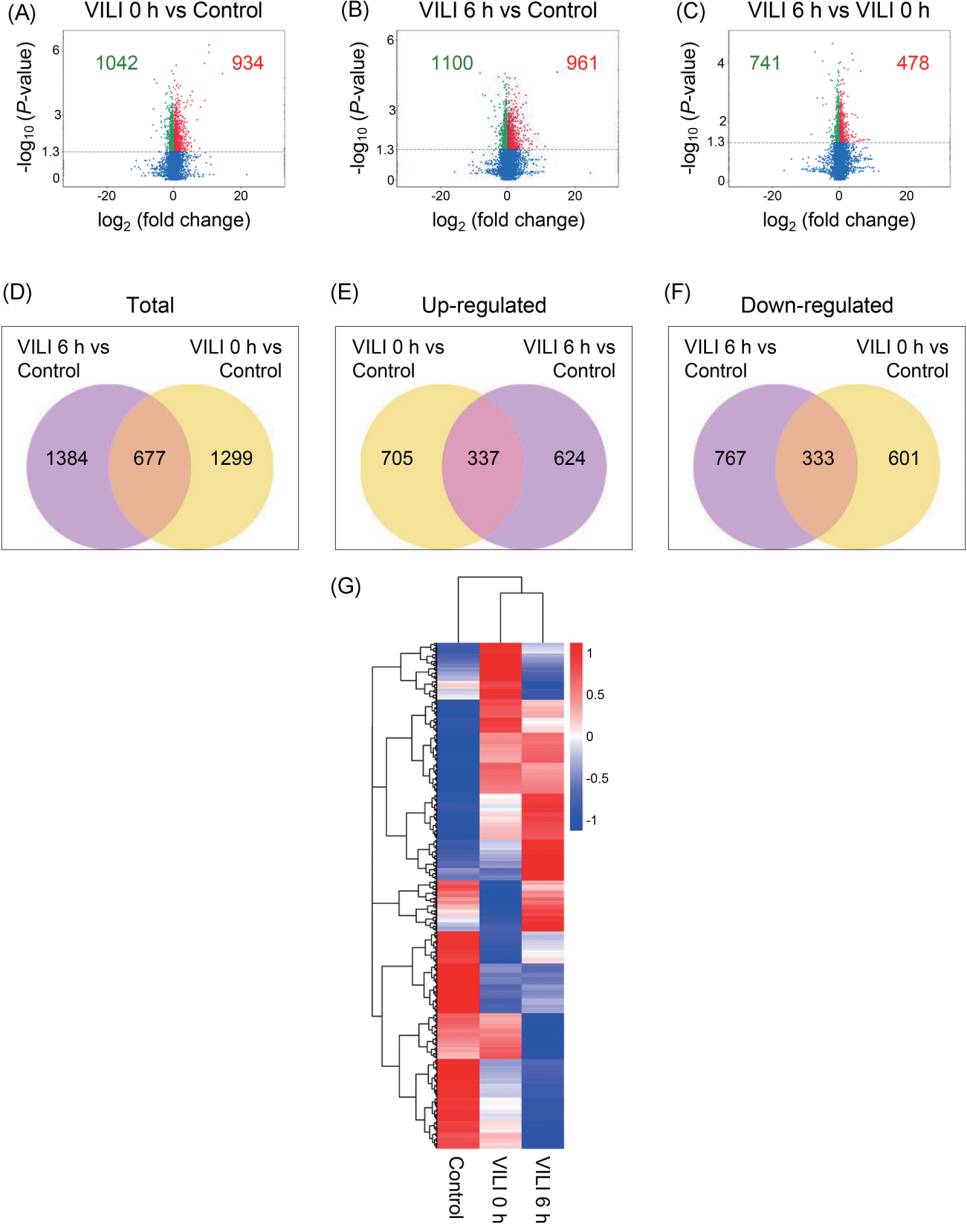
Transcriptome profiling of mRNAs in VILI. A‐C, Volcano plot for the differentially expressed mRNAs in the comparisons of VILI 0 hours vs control, VILI 6 hours vs control, and VILI 6 hours vs VILI 0 hours, respectively. Red represents upregulated transcripts and green represents downregulated transcripts. Blue denotes diﬀerentially expressed transcripts that did not pass the significance threshold of *P* < 0.05. D‐F, Total, upregulated, and downregulated mRNAs showing the overlapping transcripts in multiple comparisons by Venn diagram, respectively. G, Heat map of the differentially expressed mRNAs. mRNA, messenger RNA; VILI, ventilator‐induced lung injury

We generated volcano plots to visualize the differential expression profiles of genes with the statistical significance threshold set at *P* < 0.05. Overall, 1976 mRNA transcripts in VILI 0 hours vs control, 2061 mRNAs in VILI 6 hours vs control, and 1219 mRNAs in VILI 6 hours vs VILI 0 hours, were found to be differentially expressed (Figure 2A‐C). To determine the similarity between the comparisons, Venn diagrams were generated. Some genes were found only in one of these comparisons, suggesting the temporary expression patterns of these genes (Figure 2D‐F**)**. A heat map of the differentially expressed mRNAs is displayed in Figure 2G.

**Figure 3:**
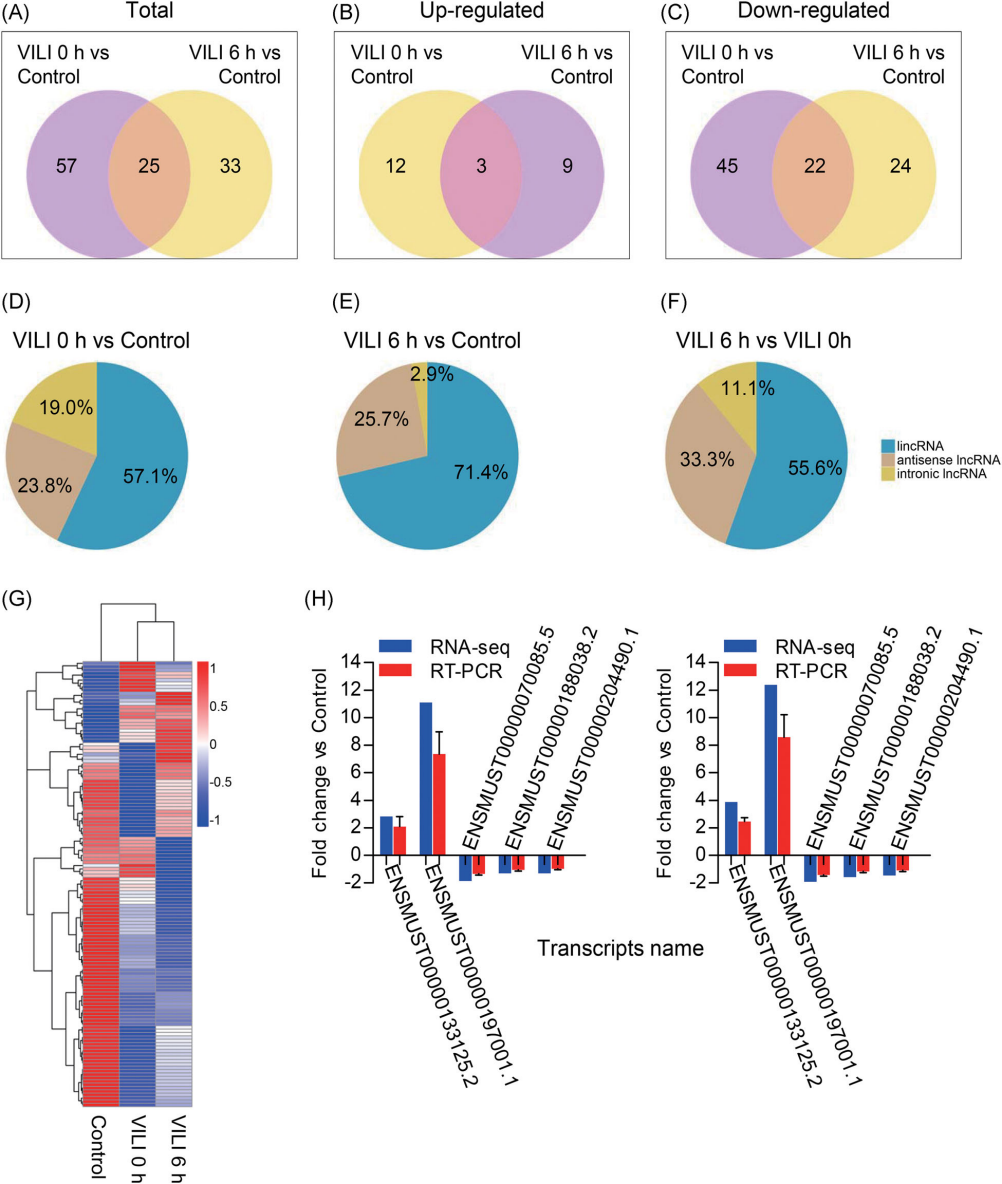
Differentially expressed profiles of lncRNAs in VILI. A‐C, Venn diagram exhibiting the total, upregulated, and downregulated lncRNAs in the comparisons. D‐F, Categories of the altered lncRNAs identified among the three comparisons by pie charts. G, Heat map of the differentially expressed lncRNAs among control, VILI 0 hours, and VILI 6 hours groups. H, Verification of the lncRNA expression levels by qRT‐PCR. lncRNA, long noncoding RNA; qRT‐PCR, quantitative real‐time polymerase chain reaction; VILI, ventilator‐induced lung injury

Importantly, many lncRNAs were found to be dysregulated. We identified 82 differentially expressed lncRNAs in VILI 0 hours vs control, 58 lncRNAs in VILI 6 hours vs control, and 32 lncRNAs in VILI 6 hours vs VILI 0 hours (Figure 3A‐C). Further, the class distribution of differentially regulated lncRNAs was analyzed, including lincRNA, antisense, and intronic transcripts. Detailed biotypes of these lncRNAs are shown in Figure 3D‐F, which shows that most of the lncRNAs were in the intergenic lncRNA category. A heat map revealed the variations in the expression of lncRNAs among groups (Figure 3G). The three groups exhibited distinct lncRNA expression profiles, suggesting different pathogenesis processes during the inflammation progression of VILI. In addition, five differentially expressed lncRNA transcripts were selected to validate the reliability of our RNA‐Seq results by qRT‐PCR, and the expression trends found by qRT‐PCR were consistent with our sequencing data (Figure 3H). Primers used are listed in Table S2. In summary, these results revealed that overstretch ventilation led to the distinct expression of many lncRNAs and mRNAs in lung tissue, implicating unique transcriptome features that provided a rationale for investigating the molecular features of VILI development.

### 3.3 Dysfunction of immune/inflammatory response and response to stress are central features of VILI

The differentially expressed genes were analyzed by GO terms to ascertain the specific categories of BPs, cellular processes (CC), and molecular function (MF) of differentially expressed transcripts. For the differentially expressed mRNAs between VILI 0 hours and the control, the most significantly upregulated and downregulated GO terms included the following BP terms: response to stress, immune system process, intracellular signal transduction, and regulation of the cellular metabolic process. The dysregulated mRNAs were associated with the adherens junction (CC), while MF terms included cytokine binding and kinase activator activity (Figure S1A and S1B). In VILI 6 hours vs control, the significantly overrepresented terms are depicted in Figure S1C and S1D. For BP analysis, the most‐enriched terms included response to stress, response to cytokine stimulus, immune system process, and cellular metabolic process; the most‐enriched CC terms included adherens junction and anchoring junction, and overrepresented MF terms included cytokine receptor activity. Comparing VILI 6 vs VILI 0 hours, results of the GO annotations collectively implicated response to stress, intracellular signal transduction, and metabolic process (BP); adherens junction, anchoring junction, cell‐cell adherens junction, and cell junction (CC); and cytokine receptor binding (MF) (Figure S1E and S1F).

We next considered these differentially expressed genes for KEGG pathway analysis, and the top‐20 pathways are summarized. In VILI 0 hours vs control, the forkhead box O (FoxO) signaling pathway, adherens junction, nuclear factor‐κB (NF‐κB) signaling pathway, and the tumor necrosis factor (TNF) signaling pathway were the most enriched (Figure 4A and 4B). Signaling pathways involved in adherens junction, TNF signaling pathway, HIF‐1 signaling pathway, NF‐κB signaling pathway, chemokine signaling pathway, and FoxO signaling pathway were among the most significantly enriched pathways in VILI 6 hours vs control (Figure 4C and 4D). The HIF‐1 signaling pathway, FoxO signaling pathway, and vascular endothelial growth factor (VEGF) signaling pathway were most enriched when comparing VILI 6 with VILI 0 hours samples (Figure 4E and 4F). The pathway patterns determined by KEGG could provide evidence for conducting further studies on the role of lncRNAs in the inflammation of VILI.

Figure 4Kyoto Encyclopedia of Genes and Genomes (KEGG) pathway enrichment analysis on mRNAs. A,B, Top‐20 of the upregulated and downregulated KEGG pathways in VILI 0 hours vs control. C,D, The predominant top‐20 upregulated and downregulated signaling pathways enriched in VILI 6 hours vs control. E,F, The increased and decreased pathways of top‐20 between VILI 6 and VILI 0 hours are exhibited, respectively. mRNA, messenger RNA; VILI, ventilator‐induced lung injury

**Figure:**
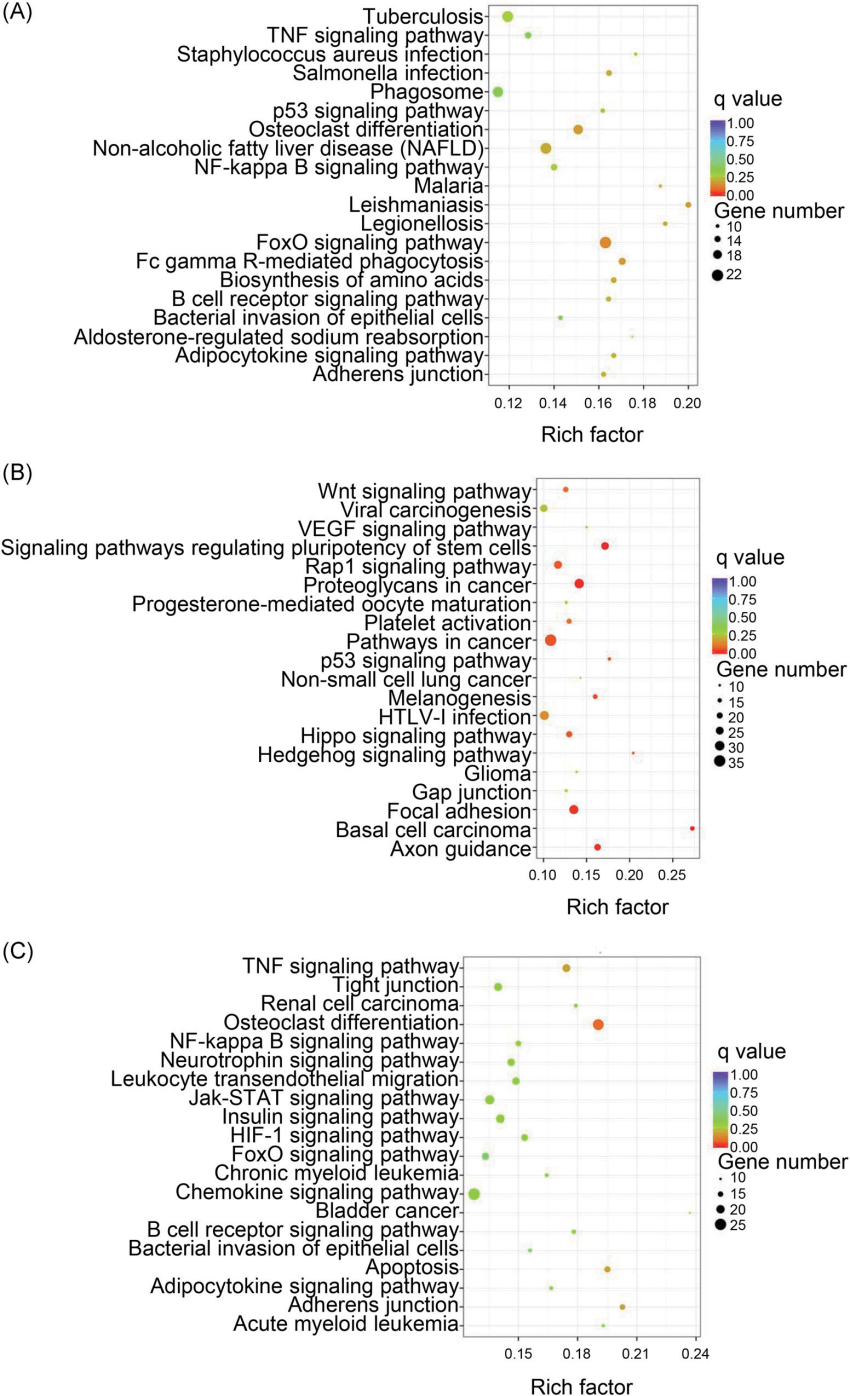

**Figure:**
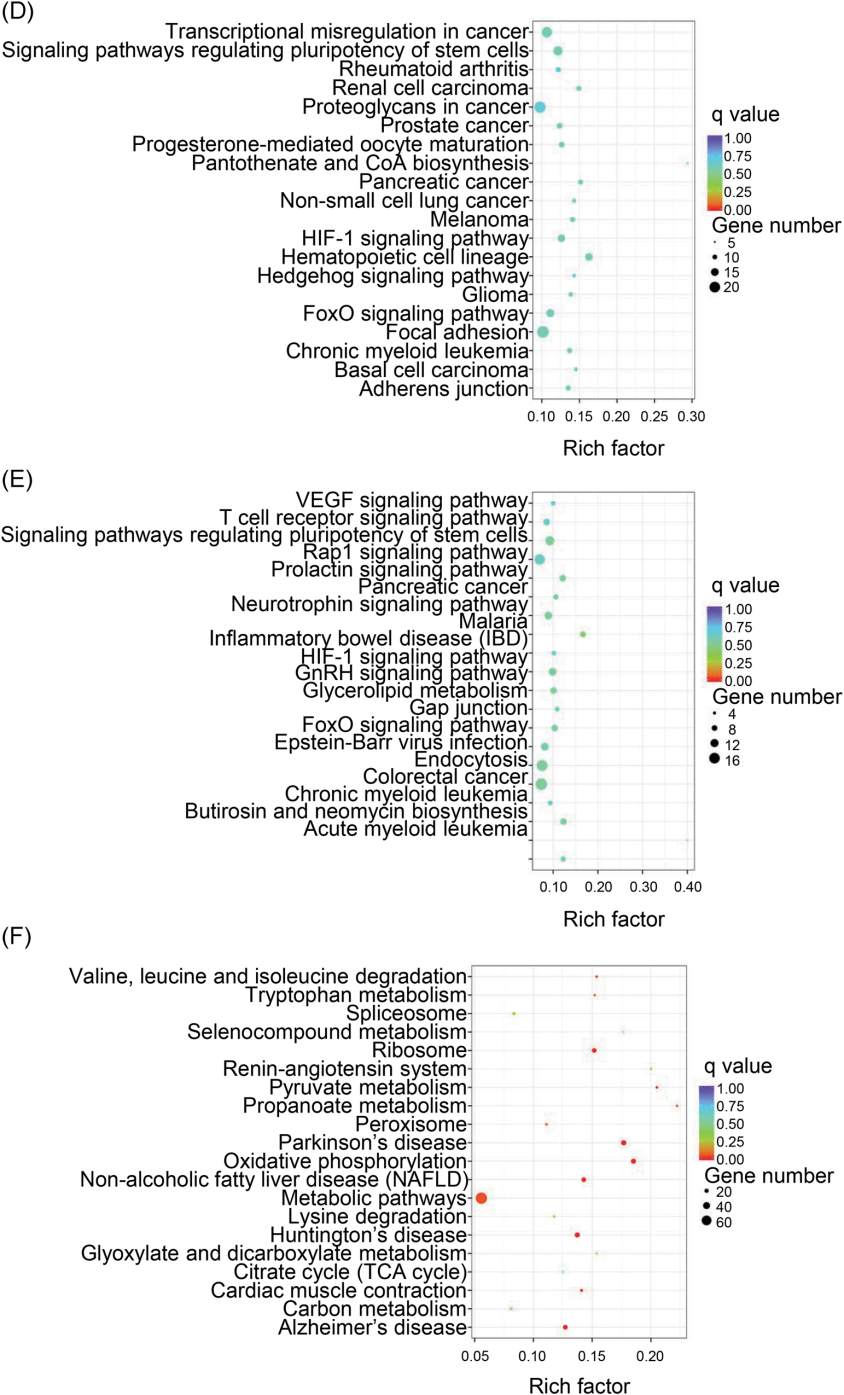

### 3.4 Overlap of VILI 0 hours and VILI 6 hours in the RNA‐seq data

**Figure 5:**
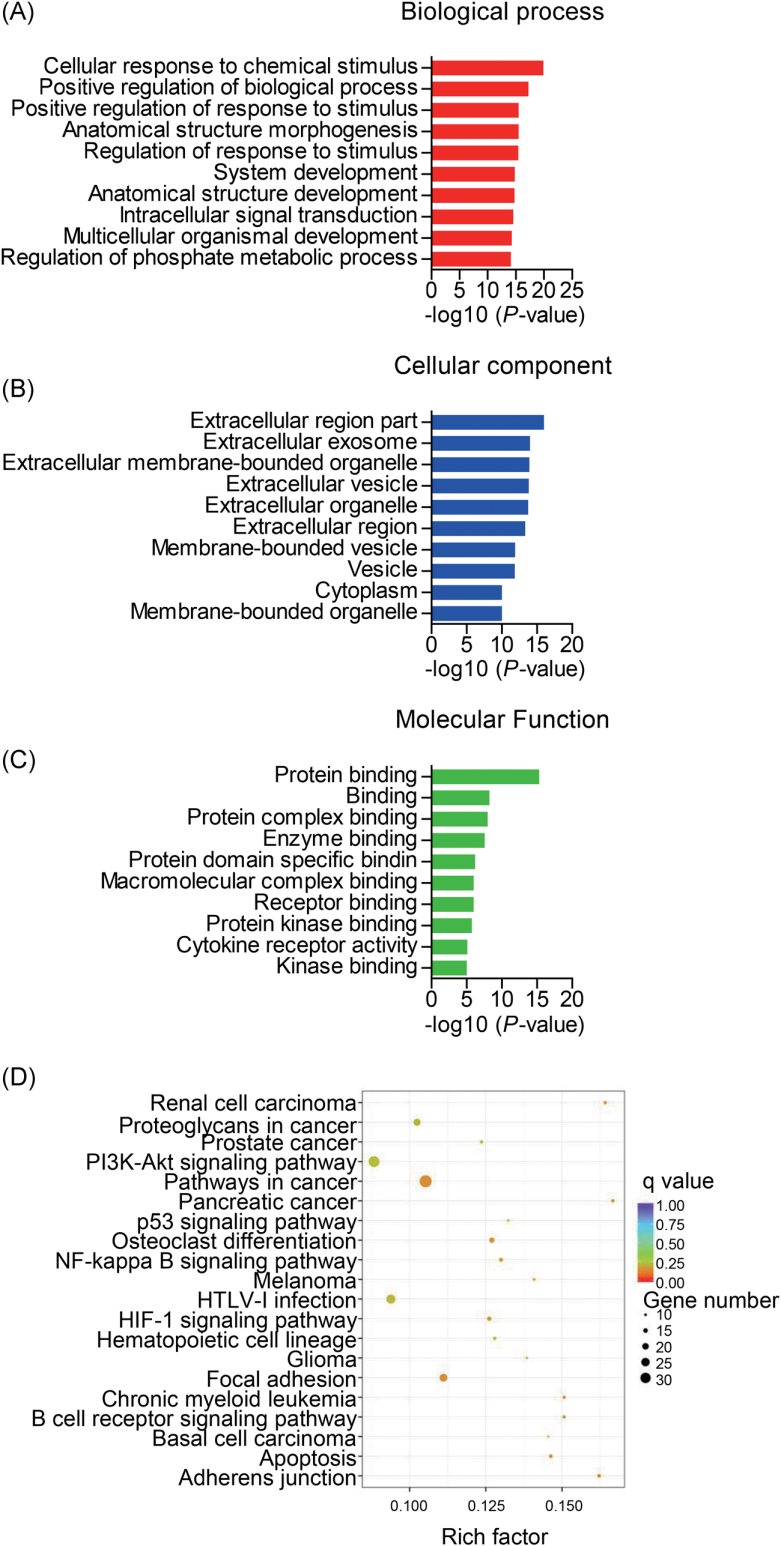
Overlap of the protein‐coding genes dysregulated upon VILI. A‐C, The enriched GO terms in top‐10 for the shared mRNAs upon VILI in 0 hours and 6 hours groups. D, Top‐20 KEGG pathways for the common mRNAs. GO, Gene Ontology; KEGG, Kyoto Encyclopedia of Genes and Genomes; mRNA, messenger RNA; NF‐κB, nuclear factor‐κB; VILI, ventilator‐induced lung injury

The altered genes in the VILI 0 hours group overlapped with genes in the VILI 6 hours group when compared with the control group. We, therefore, explored the common mRNAs obtained from these two comparisons to better determine the pathophysiological features of VILI. As indicated, we observed 677 commonly dysregulated mRNAs of the 1976 VILI 0 hours and 2061 VILI 6 hours compared with the control group, indicating their potential implications in VILI (Figure 2D‐F). These shared mRNAs were associated with core GO terms such as response to a stimulus, intracellular signal transduction, and regulation of phosphate metabolic process (BP); adherens junction and focal adhesion (CC); and cytokine receptor activity, cytokine binding, and chemokine binding (MF) (Figure 5A‐C). Further, the HIF‐1, NF‐κB, and PI3K‐Akt signaling pathways were enriched in the top‐20 during the pathway analysis (Figure 5D), indicating their consistent roles in VILI‐induced inflammation. The overlapped mRNAs suggested a model of VILI based on the alterations in the inflammation and immune response; further indicating the transcript alterations are likely to have significant implications in lung injury induced by high stretch.

### 3.5 PPI networks in VILI

**Figure 6:**
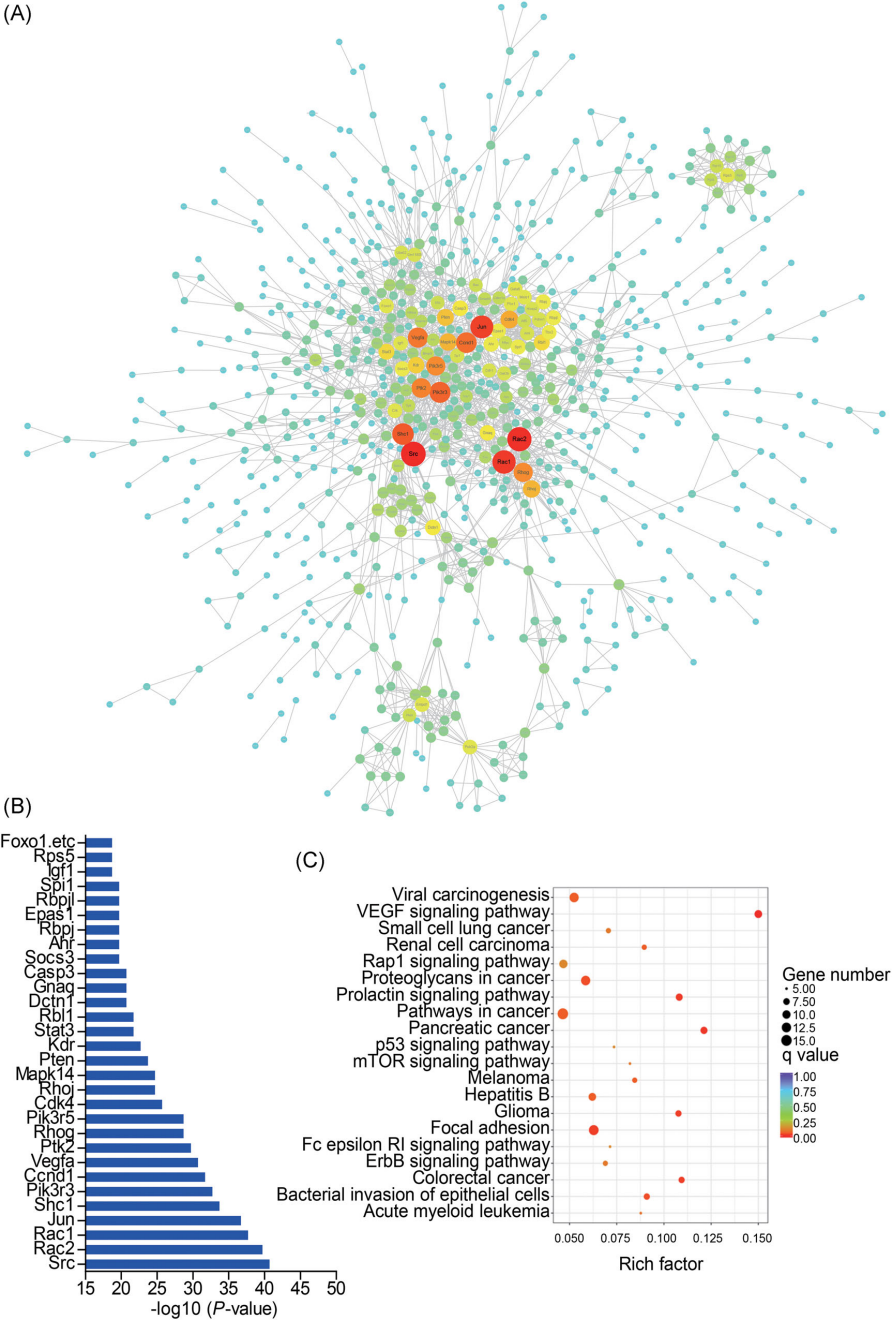
Protein‐protein interaction (PPI) networks in VILI 0 hours vs control. A, PPI networks constructed by significantly differentially expressed mRNAs between VILI 0 hours and control (*P* < 0.05). Genes are displayed as nodes and interaction as edges. The node size represents the node degree. B, List of the top‐30 hub genes with the degree of nodes. C, Top‐20 pathways of the hub genes. mRNA, messenger RNA; mTOR, mammalian target of rapamycin; VEGF, vascular endothelial growth factor; VILI, ventilator‐induced lung injury

PPI networks were constructed to analyze protein interactions between the dysregulated protein‐coding genes. The PPI network consisted of 787 nodes and 2059 edges in VILI 0 hours vs control (Figure 6A) and 839 nodes and 1900 edges in VILI 6 hours vs control (Figure S2A). The node degree was applied to evaluate the crucial roles of genes and the top‐30 genes with the highest connection degree changes are shown within networks (Figure 6B; Figure S2B). Furthermore, the top‐30 hub genes were examined by KEGG pathway analysis. These genes were mainly enriched in the VEGF signaling pathway, focal adhesion, and bacterial invasion of epithelial cells in the VILI 0 hours vs control (Figure 6C), and the FoxO signaling pathway, chemokine signaling pathway, and focal adhesion in VILI 6 hours vs control (Figure S2C).

### 3.6 Functional analysis of differentially expressed lncRNAs

Importantly, lncRNAs play a role in regulating mRNA expression in *trans* and in *cis*, contributing to their possible regulatory mechanisms. We consequently explored the target genes of the differentially expressed lncRNAs identified in this study. To identify potential target genes, we set a threshold of colocation as 100 kb upstream and 100 kb downstream of each lncRNA. Some mRNAs were found that could be potentially affected by the modified lncRNAs in *cis*, but there were very few significant enriched GO terms detected.

Next, the *trans*‐regulated mRNAs for the altered lncRNAs were predicted. lncRNA‐mRNA pairs were built to facilitate their internal relationship. This coexpression network encompassed 81 lncRNA nodes, 835 mRNA nodes, and 2636 edges in VILI 0 hours vs control (Figure S3), and 1637 edges linking 55 lncRNA nodes, 655 mRNA nodes in the network of VILI 6 hours vs control (Figure S4). The lncRNA‐mRNA coexpression networks could help better integrate the coexpressed mRNAs with the regulatory functions of lncRNAs.

**Figure 7:**
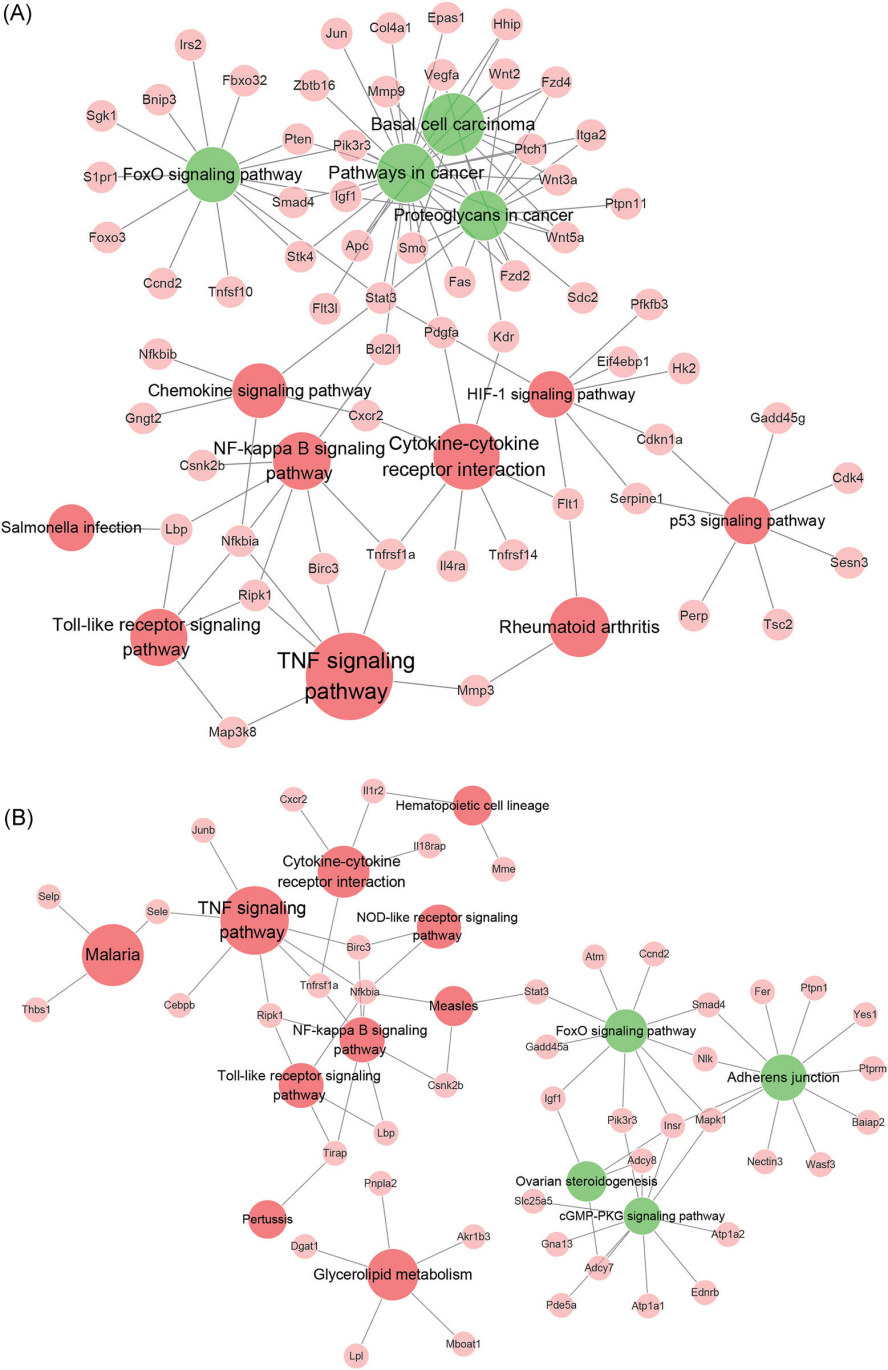
Kyoto Encyclopedia of Genes and Genomes (KEGG) pathway enrichment analysis of altered lncRNAs and coexpressed genes. A, The most‐enriched KEGG pathways of the differentially expressed lncRNAs and coexpressed mRNAs in VILI 0 hours vs control. B, The predominant enriched pathways characterized by the dysregulated lncRNAs and their targeted genes of VILI 6 hours vs control. The red circles represent the upregulated pathways of the lncRNAs; green circles, downregulated pathways of the lncRNAs; pink circles, genes coexpressed with lncRNAs; edges, the connections between the pathways and related genes. The size of circles denotes the enrichment scores of the KEGG pathways. FoxO, forkhead box O; lncRNA, long noncoding RNA; mRNA, messenger RNA; NF‐κB, nuclear factor‐κB; TNF, tumor necrosis factor; VILI, ventilator‐induced lung injury

Next, the dysregulated lncRNAs were analyzed functionally. Based on the GO analysis of the coexpressed genes, a variety of biological terms correlated with the lncRNAs in VILI 0 hours vs control (Figure S5A and S5B) and VILI 6 hours vs control (Figure S5C and S5D), including response to stress, inflammatory response, cellular response to cytokine stimulus, intracellular signal transduction, and immune system process (BP); extracellular space (CC); and cytokine activity and chemokine activity (MF). Simultaneously, the altered lncRNAs were enriched by pathway analysis, and the most‐enriched pathways ranked according to their enrichment scores are represented in Figure 7A and 7B. Among these enriched pathways, the FoxO, NF‐κB, TNF, and chemokine signaling pathways also appeared in the mRNA pathway analysis, demonstrating a close correlation of these pathways and the effects of high‐stretch MV. Overall, our functional analysis of lncRNAs suggested that the differentially expressed lncRNAs that were also predicted to be linked with mRNA expression are a molecular component of the inflammatory characteristics of VILI, providing us with insight into their regulatory function in VILI.

### 3.7 The lncRNA Gm43181 involved in neutrophil activation is significantly correlated with lung injury severity

Notably, comparison of lncRNAs profiles from VILI 0 and VILI 6 hours uncovered 25 shared lncRNAs when compared with the control (Figure 3A‐C). We, therefore, extracted all the overlapping lncRNAs with a threshold of fold change greater than two and constructed coexpression subnetworks with the coexpressed mRNAs of fold change greater than two (Figure 8A). The subnetwork contained 10 lncRNAs nodes, 52 mRNAs nodes, and 118 connection edges, which might be the key lncRNAs involved in VILI. The expression of the top‐10 shared lncRNAs visualized in the subnetwork is shown in Figure 8B.

Figure 8Function predictions of the lncRNA Gm43181. A, Coexpression networks of the shared lncRNAs and mRNAs with fold change greater than two in both comparisons of VILI 0 hours vs control and VILI 6 hours vs control. The mRNAs are shown with green circles, lncRNAs are shown with red diamonds, and the lines denote the correlational relationship. The size represents the node degree. B, The top‐10 common lncRNAs with fold change greater than two. C,D, The coexpressed mRNAs with lncRNA Gm43181 in VILI 0 hours vs control and VILI 6 hours vs control, respectively. E,F, Gene Ontology (GO) annotations of lncRNA Gm43181 and the coexpressed mRNAs in VILI 0 hours vs control, and VILI 6 hours vs control. G, The correlation analysis between lncRNA Gm43181 expression and the lung injury score determined through qRT‐PCR. The correlation analysis between lncRNA Gm43181 and CXCR2 expression levels in lung samples determined using RNA‐Seq (H) and qRT‐PCR (I), respectively. J, Expression levels of lncRNA Gm43181 and CXCR2 in neutrophils quantified using qRT‐PCR. K, A scatterplot of lncRNA Gm43181 and CXCR2 expression levels in neutrophils determined by qRT‐PCR. *P* < 0.05 represents a significant difference. CXCR2, chemokine receptor chemokine (C‐X‐C) receptor 2; lncRNA, long noncoding RNA; qRT‐PCR, quantitative real‐time polymerase chain reaction; VILI, ventilator‐induced lung injury

**Figure:**
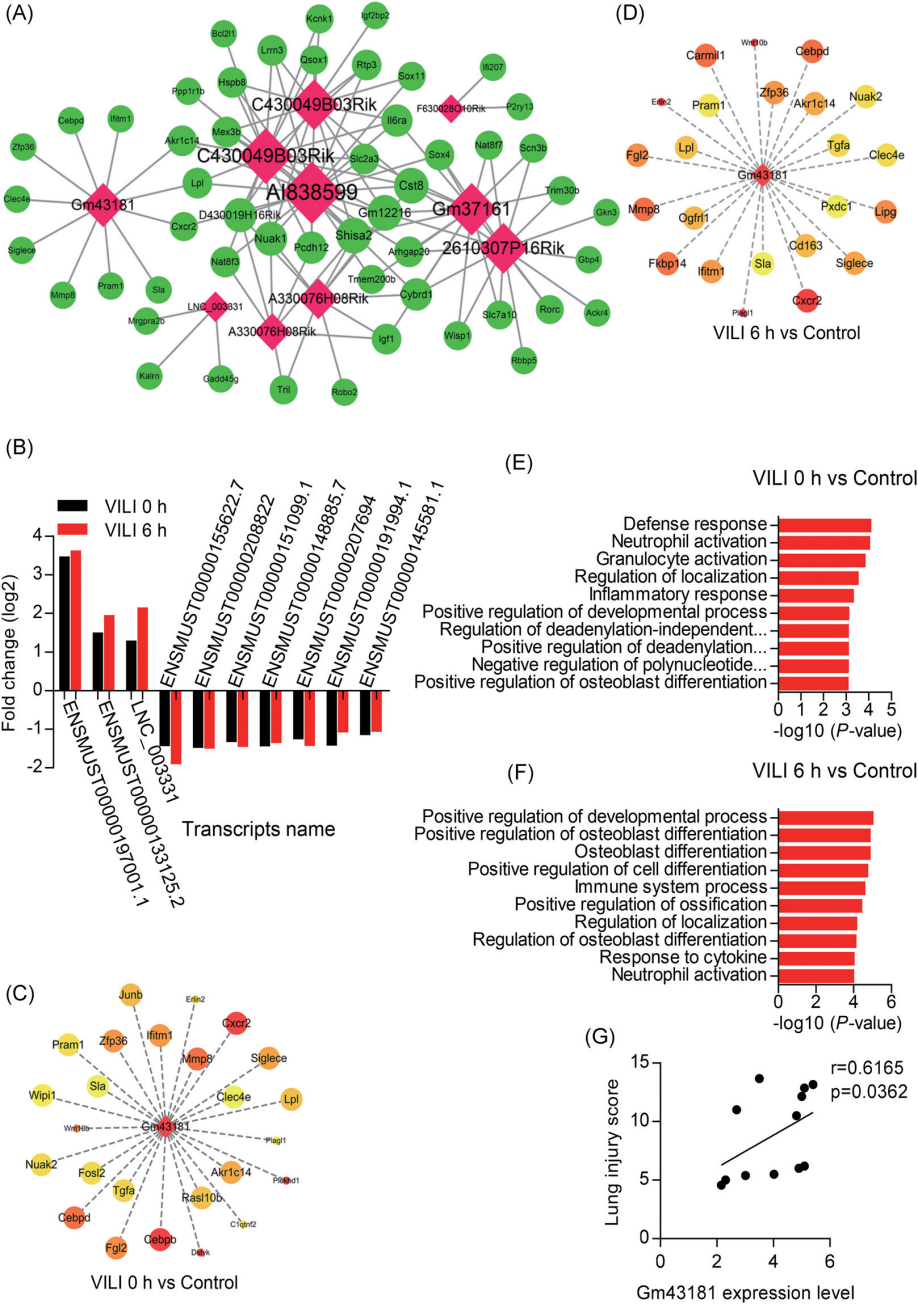

**Figure:**
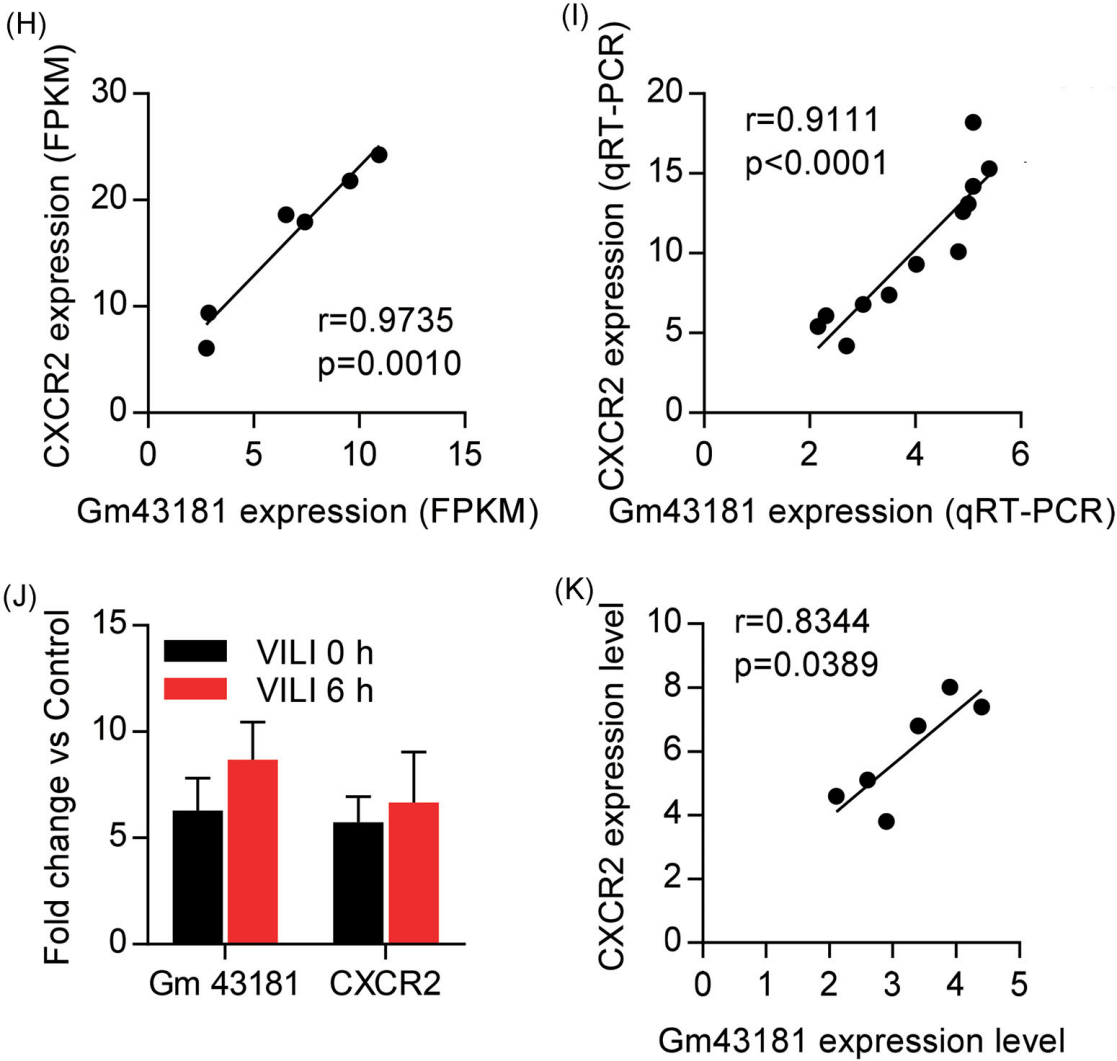

Of these common lncRNAs, we focused on the lncRNA named Gm43181 (ENSMUST00000197001.1), which was not only overexpressed but also had a relatively high node degree in the subnetwork. We, therefore, established a coexpression subnetwork between lncRNA Gm43181 and the coexpressed genes (Figure 8C and 8D). Moreover, neutrophil activation was found to be related with the lncRNA Gm43181 as annotated by GO enrichment (Figure 8E and 8F). Therefore, to determine whether there was any correlation between lncRNA Gm43181 and lung injury we conducted a Pearson correlation analysis. A positive correlation between lncRNA Gm43181 expression and the lung injury score was observed in qRT‐PCR results using an independent group of VILI models (Figure 8G). Thereafter, lncRNA Gm43181 highly correlated with its *trans*‐regulated target chemokine receptor chemokine (C‐X‐C) receptor 2 (CXCR2) determined by RNA‐Seq data (*r* = 0.9735, *P* = 0.0010) (Figure 8H), and their relationship was confirmed in independent groups of VILI mice using lung samples by qRT‐PCR (*r* = 0.9111, *P* < 0.0001) (Figure 8I). Given these results, we speculate that this lncRNA mediates the coexpression of CXCR2 on neutrophils, potentially contributing to neutrophil activation to promote VILI inflammation. Additional studies are needed to help explore the regulatory mechanism of the VILI‐associated lncRNA Gm43181, which may be identified as a promising lncRNA biomarker or therapeutic target in VILI. In consequence, some experiments were taken to analyze the role of lncRNA Gm43181 in VILI. We, therefore, sorted neutrophils from lungs of control, VILI 0 hours, and VILI 6 hours mice using FACS sorting and quantified the expression levels of lncRNA Gm43181 and CXCR2 in neutrophils using qRT‐PCR. As shown in Figure 8J, the expression levels of lncRNA Gm43181 and CXCR2 in neutrophils were significantly upregulated either in VILI 0 hours or VILI 6 hour groups. Moreover, a positive correlation between the expression levels of lncRNA Gm43181 on neutrophils and CXCR2 was observed (*r* = 0.8344, *P* = 0.0389) (Figure 8K).

## 4 DISCUSSION

Using RNA‐seq, we performed a comprehensive genome‐wide transcriptional screening of the regulated lncRNAs and mRNAs in the inflammatory progression of VILI model. Based on bioinformatics analysis, the dysregulated lncRNAs were mostly involved in response to stress and immune response/inflammatory processes and inflammatory pathways. Importantly, the lncRNA Gm43181 might be linked to the biological process associated with CXCR2‐mediated neutrophil activation during the inflammatory response of VILI. Altogether, our results implicated several lncRNAs to have potential regulatory roles in mediating inflammation in high‐stretch ventilation‐induced pathologies.

Previous profiling studies suggested that alterations of distinct gene expression might underlie the potential pathologies in VILI. For instance, protein‐coding genes were altered in the lungs of VILI animal model.^10^, ^11^ Interestingly, although these differentially expressed genes were associated with developmental processes, morphogenesis, metabolism, cell survival, or communication, most of them showed the same gene expression patterns like that in the inflammatory response. Additionally, noncoding RNAs such as miRNAs were also found to be dysregulated in VILI; analysis revealed that the altered miRNAs were mostly involved in inflammatory and TGF‐signaling networks.^14^ In our present study, we analyzed the transcripts in VILI 0 hours and VILI 6 hours, representing early and late stages of VILI progression, respectively. The results demonstrated that exposure to high‐stretch ventilation changed the expression patterns not only of mRNAs but also of lncRNAs in the lung at both early and relatively late time points of VILI. For dozens of the differentially expressed genes among the multiple comparisons, functional enrichment analysis implicated these genes to be associated with most of the characteristics related with VILI pathologies, including response to stress, cell metabolism, and most significantly, inﬂammatory response, immune response, and cytokine/chemokine interactions. In addition, our analysis also presented that the commonly dysregulated mRNAs upon VILI at 0 hours and VILI 6 hours were also implicated in the process of inﬂammatory and immune response. Our data, therefore, are consistent with results of previous studies that focused on the differentially expressed genes associated with an inflammatory response as the biomarker of VILI pathologies.

Several lncRNAs are reported to regulate the molecular mechanisms underlying many respiratory diseases;^20^, ^21^ however, their potential functions remain largely unclear. We identified many dysregulated lncRNAs in VILI at 0 hours and VILI 6 hours compared with control mice and analyzed them functionally. Given these results, our study not only broadens the understanding of the protein‐coding gene expression in VILI but also incorporated the regulatory roles of lncRNAs in the inflammatory course of VILI.

Dysfunction of cell apoptosis/differentiation, oxygen stress, inflammatory response, and autophagy are considered to be the underlying pathologies of VILI.^35^, ^36^, ^37^ The inflammatory response is thought to be one of the key contributing components in the molecular mechanism of VILI. Exposure to high tidal volume ventilation could activate the inflammasome or release inflammatory mediators.^3^, ^4^, ^5^, ^6^ Inhibiting or reducing inflammation was reported to have a protective role in VILI.^7^, ^8^, ^9^ Transcriptome‐profiling studies have also suggested that VILI pathology is related to inflammation.^38^, ^39^ Many inflammation‐related genes were upregulated in the lung samples of VILI animals and in the blood of patients with ventilator‐associated pneumonia.^10^, ^12^

Our data are consistent with the current comprehension of the mechanism underlying these pathologies. Importantly, growing studies have demonstrated the important role of lncRNAs in regulating the inflammatory process;^40^, ^41^ however, studies on lncRNAs in VILI are still limited to date. Furthermore, there is evidence that the regulatory roles of lncRNAs could be predicted by mRNA expression in *trans* and *cis*.^15^ Our work clearly analyzed the regulatory roles of lncRNAs by their coexpressed genes in the context of VILI. The majority of the altered lncRNAs were potentially related to stress response, immune system/inflammatory processes, and associated inflammatory pathways, which were in line with the above description of stretch‐induced inflammation. The lncRNA‐mRNA coexpression subnetworks constructed by all the overlapping lncRNAs with a fold change greater than two reveal the potential key lncRNAs involved in VILI. By comparing their expression in the lung samples obtained from mice after VILI 0 hours and VILI 6 hours, all dysregulated lncRNAs showed consistent expression patterns in these two groups. This demonstrated that these lncRNAs might exert vital roles in VILI, particularly inflammation‐mediated dysfunctions, which are worth studying further.

Our present results identified the transcriptome profiles including mRNAs and lncRNAs using RNA‐Seq, which was a useful method for detecting novel genes and transcripts that may not have been identified using a microarray technique. Unlike previous microarray analyses concentrating on protein‐coding genes,^10^, ^12^ the current study also included lncRNA function. Of relevance, Xu et al^22^ recently reported a catalog of 104 lncRNAs and 809 mRNAs that are differentially expressed as measured by RNA‐Seq in the lung samples of a rat VILI model compared with sham control. When compared with data from Xu's study, our study sequenced not only VILI 0 hours and control but also VILI 6 hours group (the lung inflammation is most severe), highlighting alterations of lncRNAs and mRNAs during early and relatively late time intervals of lung inflammation systematically. However, in our RNA‐Seq data, the numbers of lncRNAs were relatively lower than those observed in Xu et al's study. This can likely be attributed to the differences in models (mouse model vs rat model), parameter settings for ventilation (tidal volume, 20 vs 30 ml/kg respiratory frequency: 40 vs 70 breaths/min), conditions (VILI 6 hours, VILI 0 hours, and control groups vs VILI and sham groups), and inclusion criteria used for the altered genes (*P* < 0.05 vs fold change > 2 and *P* < 0.05). Despite these differences, the list of differentially expressed lncRNAs in our study along with the data from Xu et al lead to the conclusion that a subset of these lncRNAs might have regulatory functions in the progression of VILI inflammation.

In our analysis, we focus on a lncRNA named Gm43181 that was overexpressed with the highest fold change in the common lncRNAs. Moreover, CXCR2 was identified as one of the coexpressed genes of this lncRNA with high expression levels both in VILI 0 hours and VILI 6 hours groups. Interestingly, the GO term annotations linked the lncRNA Gm43181 to the bioprocess of neutrophil activation. CXCR2 is a chemokine receptor belonging to CXCR chemokine family that is activated by chemokine (C‐X‐C motif) ligand 1 and 2/3 (CXCL1 and CXCL2/3), and it is expressed on neutrophils, where its activation could lead to neutrophil recruitment.^42^ Correspondingly, previous studies have suggested that neutrophil activation and infiltration play a central role in contributing to VILI inflammation.^43^, ^44^, ^45^, ^46^ The formation of extensive hyaline membranes, inflammatory mediator production, and increased permeability of the alveolar‐capillary barrier caused by mechanical ventilation challenge was reduced in neutrophil‐depleted or‐inhibited animals.^44^, ^45^, ^46^ Therefore, the lncRNA Gm43181 may promote inflammation by regulating the recruitment of neutrophils into the lung through the overexpression CXCR2. Thus, our results agreed with those of previous studies that CXCR2 might be a vital mediator of lung injury.^47^, ^48^ Notably, the overexpressed lncRNA Gm43181 level was positively associated with the severity of lung injury in VILI, suggesting that this lncRNA might be a potential biomarker for VILI. Additionally, the close relationship between lncRNA Gm43181 and CXCR2 in neutrophils were validated in some experiments. Accordingly, additional studies are needed to clarify lncRNA‐mediated roles in modulating neutrophils and determine the possible lncRNA Gm43181‐CXCR2 mechanisms.

Despite the significance of our findings, our study also had some limitations. On one hand, our sample size was relatively small with only three mice in each group. Further, being largely a descriptive study, we could not ascertain whether the identified lncRNAs were causing the inflammation in VILI. Moreover, as the lncRNAs' functions were determined based on bioinformatics predictions, the RNA‐Seq data should be confirmed with direct evidence from subsequent in vivo experiments. The short lifespan of neutrophils restricted the study of the role of lncRNA Gm43181. Nonetheless, these observations provide a basis for the transcriptome signatures in the lung inflammation during VILI progression.

Collectively, our study might set the stage for better understanding the pathogenesis and precise manipulation of VILI as it relates to lncRNAs, and further studies are needed to help delineate the mechanism of lncRNAs in regulating inflammation during VILI.

## CONFLICT OF INTERESTS

The authors declare that there are no conflict of interests.

## Supporting information

Supplementary information

Supplementary information

Supplementary information

Supplementary information

Supplementary information

Supplementary information

Supplementary information

Supplementary information

Supplementary information
