# Supplementary figures and images for: Expression profiling analysis of long noncoding RNAs in a mouse model of ventilator‐induced lung injury indicating potential roles in inflammation
Source: J Cell Biochem. 2019 Feb 19;120(7):11660–79. doi: 10.1002/jcb.28446 (PMC7983175; doi:10.1002/jcb.28446)

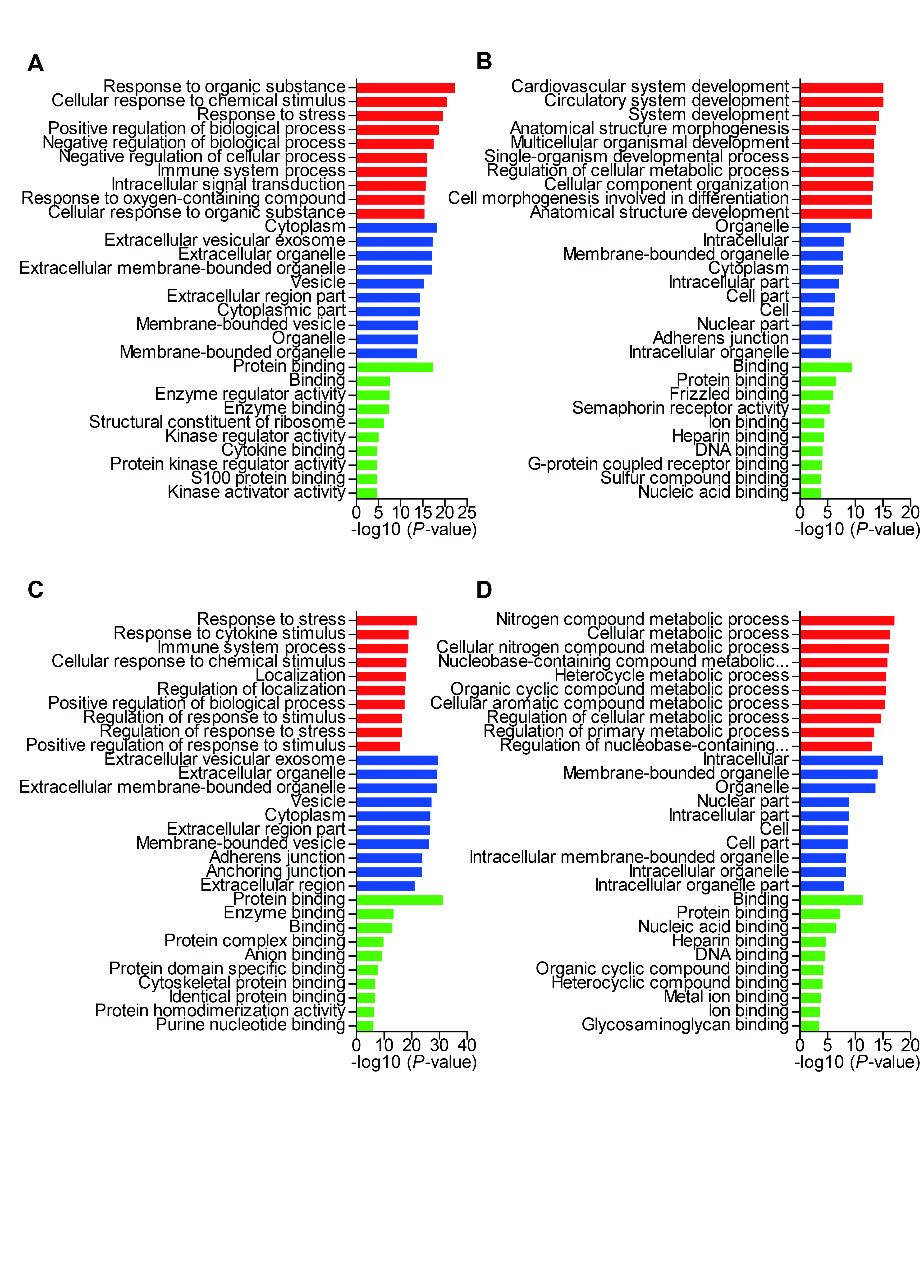

Supplement: Supplementary file 1 — Supplementary information [file JCB-120-11660-s006.tif]

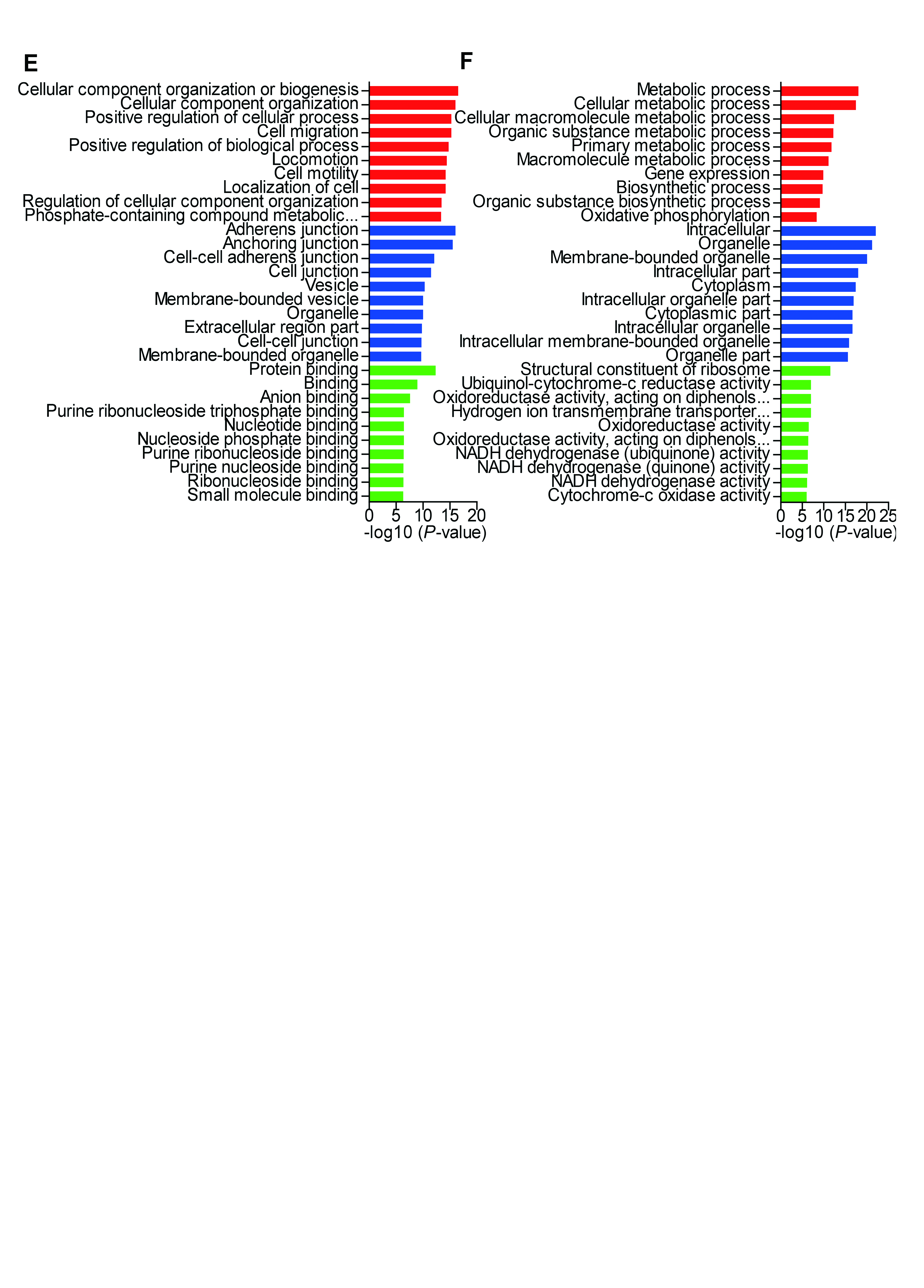

Supplement: Supplementary file 2 — Supplementary information [file JCB-120-11660-s005.tif]

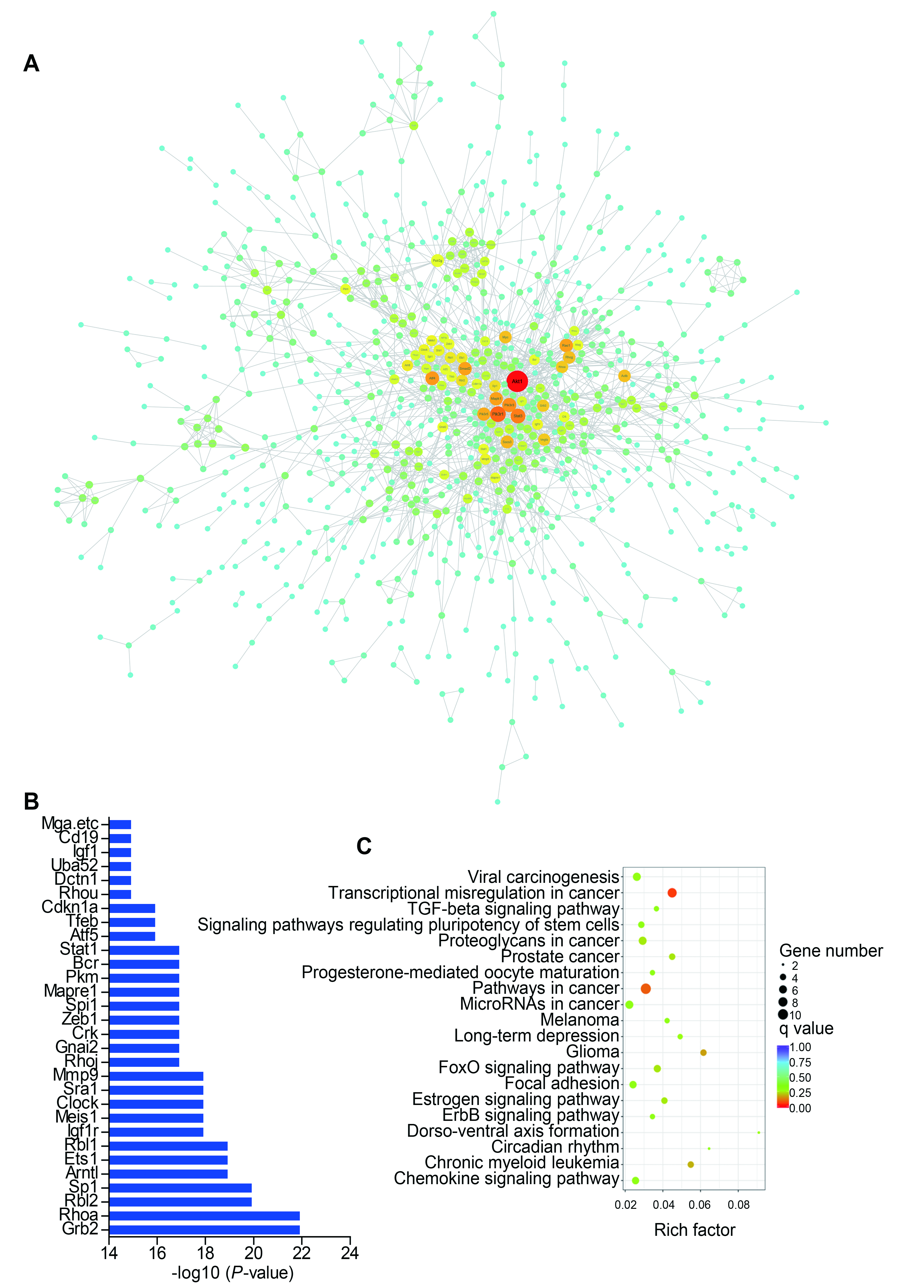

Supplement: Supplementary file 3 — Supplementary information [file JCB-120-11660-s004.tif]

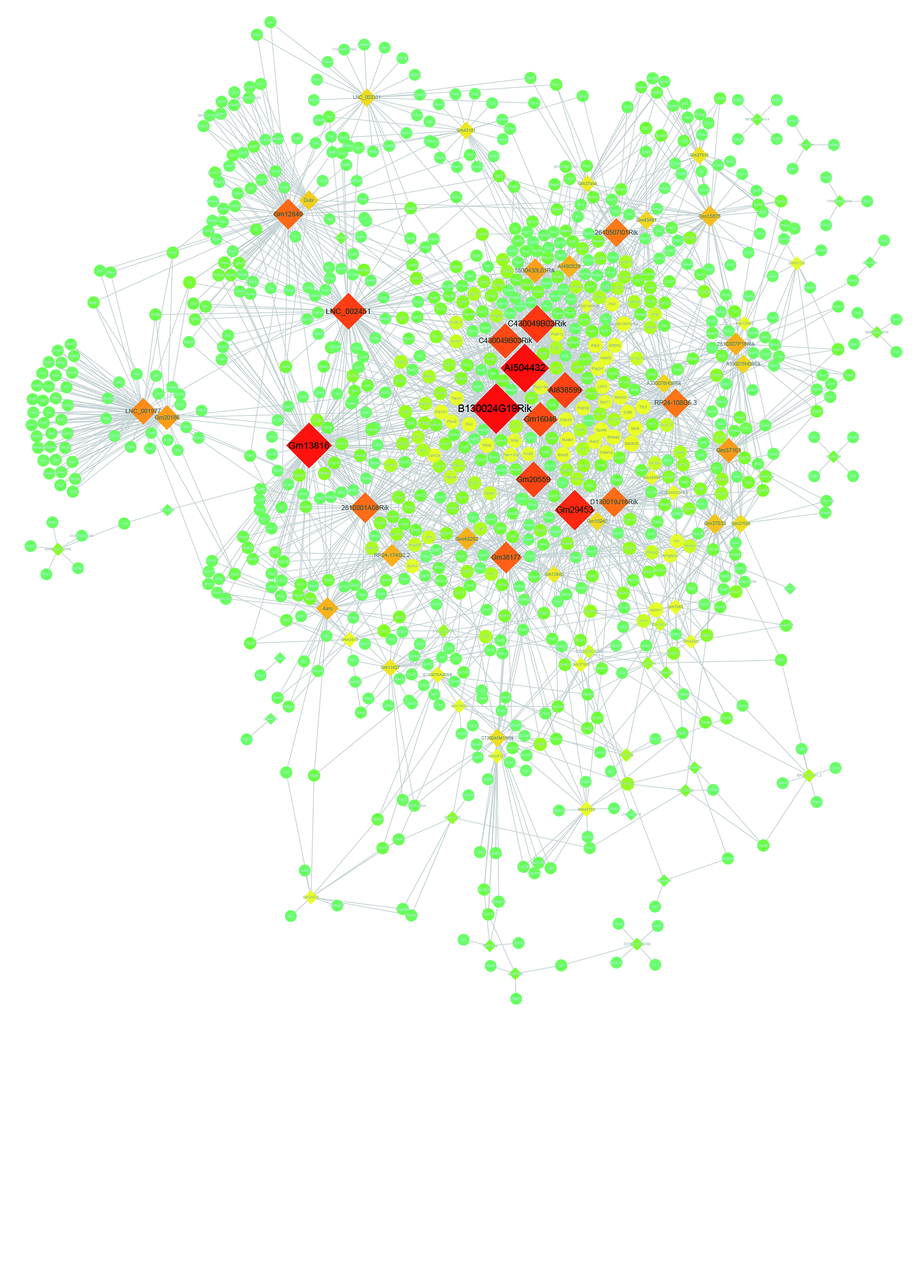

Supplement: Supplementary file 4 — Supplementary information [file JCB-120-11660-s008.tif]

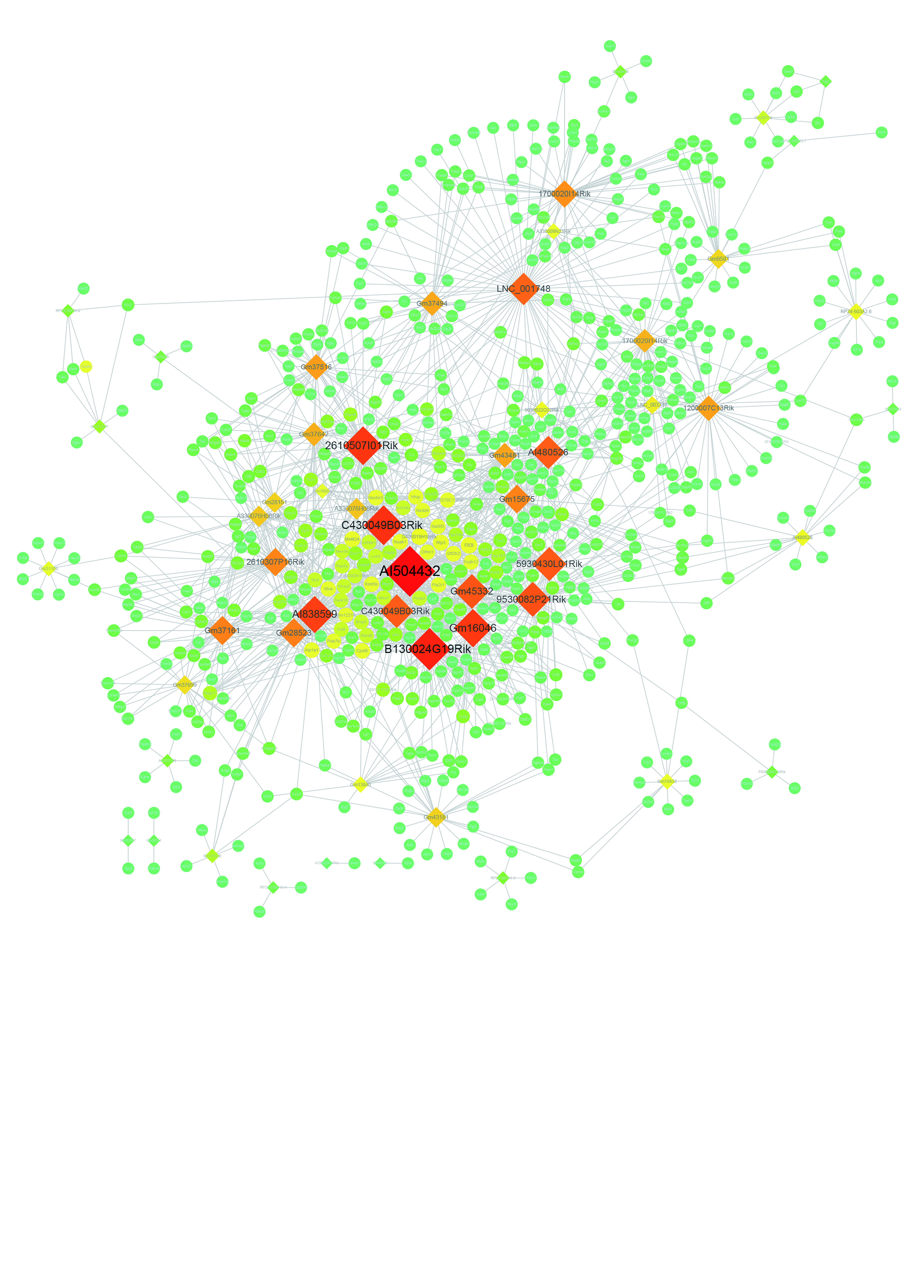

Supplement: Supplementary file 5 — Supplementary information [file JCB-120-11660-s001.tif]

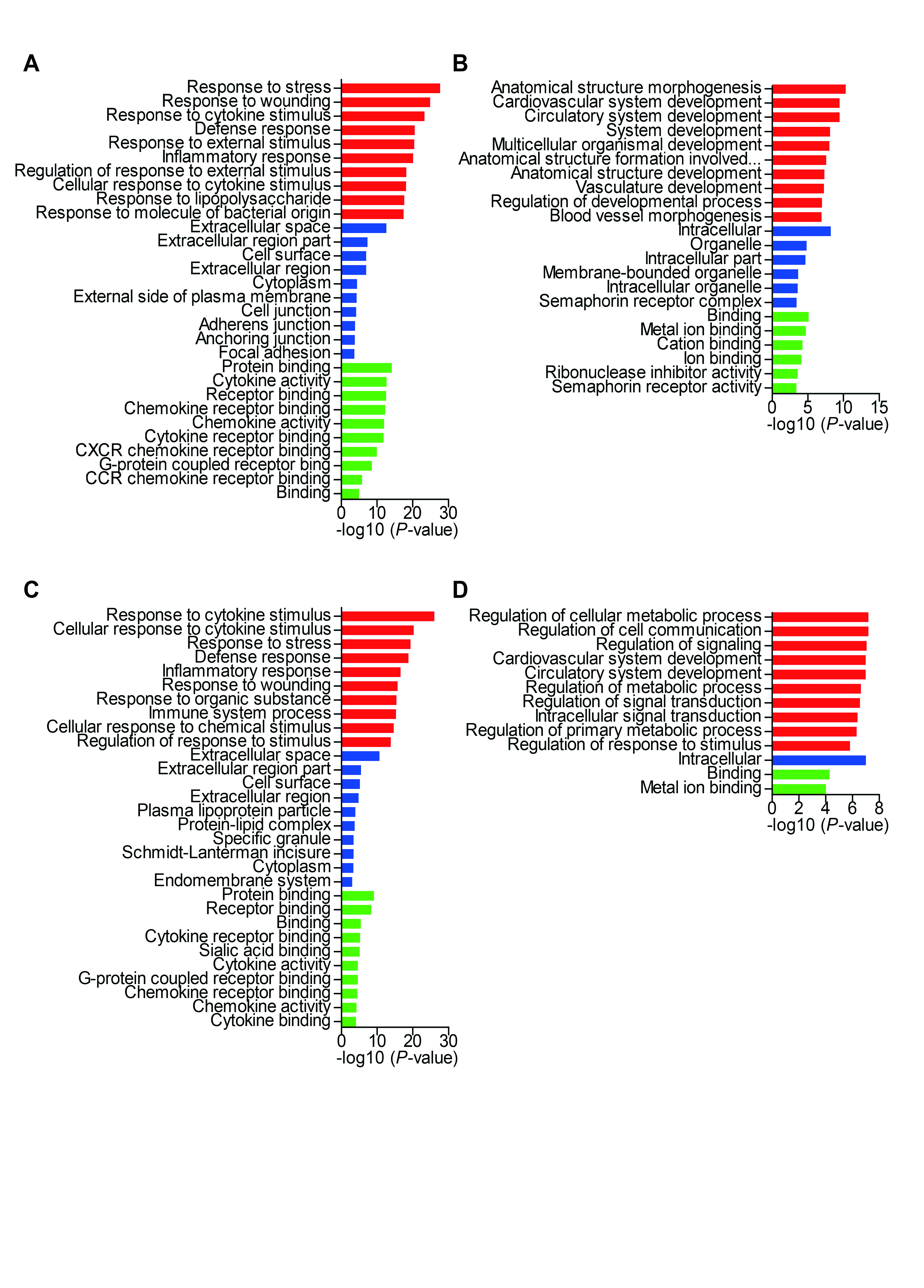

Supplement: Supplementary file 6 — Supplementary information [file JCB-120-11660-s002.tif]
