# Supplementary material for: Expression profiling analysis of long noncoding RNAs in a mouse model of ventilator‐induced lung injury indicating potential roles in inflammation
Source: J Cell Biochem. 2019 Feb 19;120(7):11660–79. doi: 10.1002/jcb.28446 (PMC7983175; doi:10.1002/jcb.28446)
Supplement: Supplementary file 7 — Supplementary information [file JCB-120-11660-s003.docx]

**Supplementary Figure legends**

**Supplementary Figure S1** The Gene Ontology (GO) analysis of the differentially expressed mRNAs dysregulated upon VILI. A-B, Analysis of the up-regulated and down-regulated GO terms of the top 10 on the differentially expressed mRNAs in VILI 0 h vs. control. C-D, Top 10 predominant increased and decreased GO annotations on the differentially expressed mRNAs in VILI 6 h vs. control. E-F, Top 10 of the up-regulated and down-regulated GO terms in VILI 6 h vs. VILI 0 h are displayed, respectively.

**Supplementary Figure S2** Protein-protein interaction (PPI) networks in VILI 6 h vs. control. A, PPI networks consisted of the differentially expressed mRNAs with *P* value <0.05 are shown. The nodes represent the genes and edges signify the integration of PPI. The different sizes and colors signify the node degree in the network. B, The 30 top degree genes involved in the network. C, The enriched pathways constituted by the hub genes.

**Supplementary Figure S3** The lncRNA-mRNA co-expression network in VILI 0h vs. control. The circles represent mRNAs; diamonds, lncRNAs; edges, correlational relationship between lncRNAs and mRNAs. The distinct sizes and colors highlight the degree of the nodes.

**Supplementary Figure S4** Visualization of the lncRNA-mRNA co-expression network in VILI 6h vs. control. The nodes of circle represent mRNAs, the diamond nodes denote lncRNAs, and edges show the connections of lncRNAs and mRNAs. The different sizes and colors signify the node degree in the network.

**Supplementary Figure S5** The Gene Ontology (GO) annotations on the desregulated lncRNAs. A-B, Top 10 increased and decreased Go annotations on the target genes of the differentially expressed lncRNAs in VILI 0 h vs. control. C-D, Analysis of the top 10 up-regulated and down-regulated GO categories of the co-expressed mRNAs of the altered lncRNAs.
