# Supplementary material for: Expression profiling analysis of long noncoding RNAs in a mouse model of ventilator‐induced lung injury indicating potential roles in inflammation
Source: J Cell Biochem. 2019 Feb 19;120(7):11660–79. doi: 10.1002/jcb.28446 (PMC7983175; doi:10.1002/jcb.28446)
Supplement: Supplementary file 8 — Supplementary information [file JCB-120-11660-s009.docx]

**Supplementary Table S1** Mapping conditions and statistics of RNA sequencing reads of each sample

| Sample name | Control_1 | Control_2 | Control_3 | VILI 0 h_1 | VILI 0 h_2 | VILI 0 h_3 | VILI 6 h_1 | VILI 6 h_2 | VILI 6 h_3 |
| --- | --- | --- | --- | --- | --- | --- | --- | --- | --- |
| Total reads | 95909676 | 91774924 | 104250242 | 98044676 | 106736558 | 115588258 | 107073994 | 86572714 | 87872490 |
| Clean reads | 95909676 | 91774924 | 104250242 | 98044676 | 106736558 | 115588258 | 107073994 | 86572714 | 87872490 |
| Total  mapped | 89340036  (93.15%) | 85683160  (93.36%) | 97598548  (93.62%) | 89915823  (91.71%) | 98346840  (92.14%) | 107456092  (92.96%) | 98594876  (92.08%) | 77065975  (89.02%) | 80774291  (91.92%) |
| Multiple  mapped | 3989805  (4.16%) | 3863868  (4.21%) | 4370112  (4.19%) | 4935265  (5.03%) | 5758262  (5.39%) | 6202543  (5.37%) | 5491522  (5.13%) | 3824185  (4.42%) | 4682443  (5.33%) |
| Uniquely  mapped | 85350231  (88.99%) | 81819292  (89.15%) | 93228436  (89.43%) | 84980558  (86.68%) | 92588578  (86.74%) | 101253549  (87.6%) | 93103354  (86.95%) | 73241790  (84.6%) | 76091848  (86.59%) |
| Reads map  to '+' strand | 42647476  (44.47%) | 40880469  (44.54%) | 46580167  (44.68%) | 42463526  (43.31%) | 46263487  (43.34%) | 50587258  (43.77%) | 46521141  (43.45%) | 36603013  (42.28%) | 38020812  (43.27%) |
| Reads map  to '-' strand | 42702755  (44.52%) | 40938823  (44.61%) | 46648269  (44.75%) | 42517032  (43.36%) | 46325091  (43.4%) | 50666291  (43.83%) | 46582213  (43.5%) | 36638777  (42.32%) | 38071036  (43.33%) |
| Non-splice  reads | 69478629  (72.44%) | 67229200  (73.25%) | 75341125  (72.27%) | 66142834  (67.46%) | 71425682  (66.92%) | 79533219  (68.81%) | 73735012  (68.86%) | 58061843  (67.07%) | 61410872  (69.89%) |
| Splice  reads | 15871602  (16.55%) | 14590092  (15.9%) | 17887311  (17.16%) | 18837724  (19.21%) | 21162896  (19.83%) | 21720330  (18.79%) | 19368342  (18.09%) | 15179947  (17.53%) | 14680976  (16.71%) |
