# Supplementary material for: Expression profiling analysis of long noncoding RNAs in a mouse model of ventilator‐induced lung injury indicating potential roles in inflammation
Source: J Cell Biochem. 2019 Feb 19;120(7):11660–79. doi: 10.1002/jcb.28446 (PMC7983175; doi:10.1002/jcb.28446)
Supplement: Supplementary file 9 — Supplementary information [file JCB-120-11660-s007.docx]

**Supplementary Table S2** Specific primers designed for selected long non-coding RNAs (lncRNAs)

| Gene id | Forward Primers (5’-3’) | Reverse Primers (5’-3’) |
| --- | --- | --- |
| ENSMUST00000133125.2 | AACAGGGGAAAAGATAGATAA | TGAATGGGTAAAACTAGATGA |
| ENSMUST00000197001.1 | TGGGGTAGTGACCTGGTTCTT | AGTGTTTGGGAGTGATTGAGC |
| ENSMUST00000070085.5 | CTGAATGGAAGGGATATGAGA | AAATGGAGAAAGAAGGGTTGT |
| ENSMUST00000188038.2 | TCTGGAGGTGGGTTTGGGTAG | ATTGAGTTGTCGGTAGTGGCG |
| ENSMUST00000204490.1 | GAAAACCTGCTTCCCACCTTA | TTTGTGCTGCTTTTCTTCCTC |
| CXCR2 | AGACACAGCTCCAGTTAGGGA | TGCTAGGATTTGAGCCTGAGT |

CXCR2, chemokine receptor chemokine [C-X-C] receptor 2
